# Towards standardized measurement of adverse events in spine surgery: conceptual model and pilot evaluation

**DOI:** 10.1186/1471-2474-7-53

**Published:** 2006-06-20

**Authors:** Sohail K Mirza, Richard A Deyo, Patrick J Heagerty, Judith A Turner, Lorri A Lee, Robert Goodkin

**Affiliations:** 1 Center for Cost and Outcomes Research, University of Washington, Seattle, Washington, USA; 2 Department of Orthopedics and Sports Medicine, University of Washington, Seattle, Washington, USA; 3 Department of Neurological Surgery, University of Washington, Seattle, Washington, USA; 4 Department of Medicine, University of Washington, Seattle, Washington, USA; 5 Department of Health Services, University of Washington, Seattle, Washington, USA; 6 Department of Biostatistics, University of Washington, Seattle, Washington, USA; 7 Department of Psychiatry and Behavioral Sciences and Department of Rehabilitation Medicine, University of Washington, Seattle, Washington, USA; 8 Department of Anesthesiology, University of Washington, Seattle, Washington, USA

## Abstract

**Background:**

Independent of efficacy, information on safety of surgical procedures is essential for informed choices. We seek to develop standardized methodology for describing the safety of spinal operations and apply these methods to study lumbar surgery. We present a conceptual model for evaluating the safety of spine surgery and describe development of tools to measure principal components of this model: (1) specifying outcome by explicit criteria for adverse event definition, mode of ascertainment, cause, severity, or preventability, and (2) quantitatively measuring predictors such as patient factors, comorbidity, severity of degenerative spine disease, and invasiveness of spine surgery.

**Methods:**

We created operational definitions for 176 adverse occurrences and established multiple mechanisms for reporting them. We developed new methods to quantify the severity of adverse occurrences, degeneration of lumbar spine, and invasiveness of spinal procedures. Using kappa statistics and intra-class correlation coefficients, we assessed agreement for the following: four reviewers independently coding etiology, preventability, and severity for 141 adverse occurrences, two observers coding lumbar spine degenerative changes in 10 selected cases, and two researchers coding invasiveness of surgery for 50 initial cases.

**Results:**

During the first six months of prospective surveillance, rigorous daily medical record reviews identified 92.6% of the adverse occurrences we recorded, and voluntary reports by providers identified 38.5% (surgeons reported 18.3%, inpatient rounding team reported 23.1%, and conferences discussed 6.1%). Trained observers had fair agreement in classifying etiology of 141 adverse occurrences into 18 categories (kappa = 0.35), but agreement was substantial (kappa ≥ 0.61) for 4 specific categories: technical error, failure in communication, systems failure, and no error. Preventability assessment had moderate agreement (mean weighted kappa = 0.44). Adverse occurrence severity rating had fair agreement (mean weighted kappa = 0.33) when using a scale based on the JCAHO Sentinel Event Policy, but agreement was substantial for severity ratings on a new 11-point numerical severity scale (ICC = 0.74). There was excellent inter-rater agreement for a lumbar degenerative disease severity score (ICC = 0.98) and an index of surgery invasiveness (ICC = 0.99).

**Conclusion:**

Composite measures of disease severity and surgery invasiveness may allow development of risk-adjusted predictive models for adverse events in spine surgery. Standard measures of adverse events and risk adjustment may also facilitate post-marketing surveillance of spinal devices, effectiveness research, and quality improvement.

## Background

An early warning system is needed to identify surgical devices and techniques that perform poorly when introduced into general practice [1]. Expensive technological innovations commonly gain widespread use based on limited comparative data and minimal systematic post-marketing surveillance [2]. Thus, awareness of adverse effects associated with these innovations accumulates haphazardly and disseminates slowly [3].

Adverse event assessment in spine surgery is mired by additional difficulties. In contrast to certain other procedures (such as hip and knee arthroplasty) that are fairly standardized across patients, spine surgery is much more individualized for the specific spinal pathology, combining various graft materials and fixation devices with varying degrees of vertebral decompression and fusion. Randomized trials of spine surgery typically focus on one or a few specific types of procedures, providing limited comparative data on the safety of different surgical approaches and devices. In observational studies, which in many ways are better suited for safety assessment [4,5], procedural variations might obscure the impact of a specific treatment. Also, the effects of treatment may differ across different groups of patients. This study was designed to develop measures and an analytical model to adjust for these variations when assessing safety of spine surgery.

We propose studying the safety of spine surgery for degenerative disease through a conceptual model in which safety is broadly defined as a function of preoperative patient, disease, and treatment characteristics:

*Therapeutic Safety *= *f*{*Patient Characteristics*|*Disease Attributes*|*Treatment Factors*}

**Figure 1:**
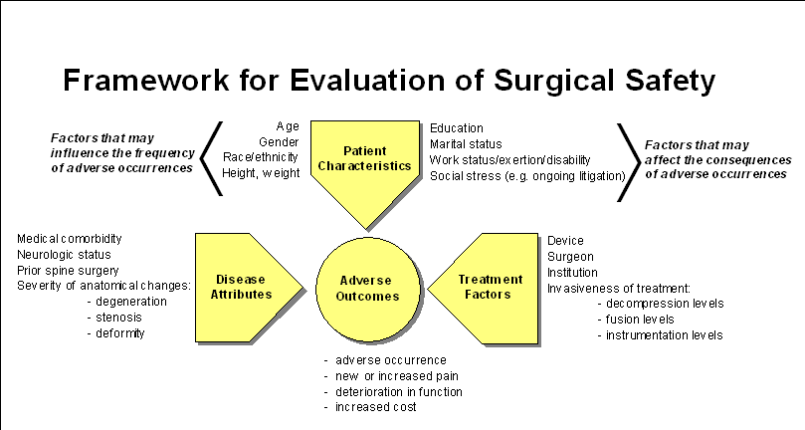
**Framework for Safety Assessment. **The relationship of patient, disease, and treatment factors to adverse outcomes.

In this framework, the effect of an individual treatment factor on safety can potentially be distinguished from the effects of other relevant patient and disease characteristics (Figure 1).

Specification of therapeutic safety is central to this model. Safety may be specified as a narrowly defined particular outcome, or it may be described as a set of adverse events characterized by specific criteria for timing, setting, severity, preventability, or causal pathway. Consistent terminology and definitions for safety outcomes are essential, both for comparing treatments and for assessing improvements over time [6].

Patient characteristics relevant for predicting surgical adverse events include age [7], height and weight (body mass index) [8], smoking status [9], burden of coexisting medical conditions [10], gender, and race [11,12]. When assessing consequences of an adverse event on clinical outcomes, such as pain or function, adjustment may also be necessary for psychosocial factors such as education, work conditions, and psychological stress [13].

To measure the severity of spinal disease, new methods are needed. Neurological function may be designated simply as normal or abnormal, or quantified by a score such as the American Spinal Injury Association (ASIA) motor score [14]. Prior surgery at the involved spinal segments may be measured as yes-no or as the number of prior operations. Quantifying degenerative structural changes across multiple spinal segments is more challenging, but at minimum, the methods must account for the severity of disc space and facet joint degeneration [15], spinal stenosis [16,17], and vertebral mal-alignment such as spondylolisthesis [12], scoliosis [18], and kyphosis [19].

New methods are also needed to measure treatment (surgical procedure) factors. Differences in the "invasiveness" of surgical procedures (e.g., route of surgical access, location of nerve roots decompressed, number of vertebrae fused and instrumented) influence risks.

The following multivariate analytical model provides a more detailed specification of the conceptual framework for evaluating the safety of spine surgery for degenerative disease:

〈AdverseOccurrence|Likelihood,orConsequences〉=β1〈PatientFactors|AgeGenderRace/ethnicity〉+β2〈CoexistingDiseaseBurden|BodyMassIndexMedical comorbidity orAnesthesia gradeSmoking status〉+β3〈SpinalDiseaseSeverity|Neuro log⁡ical functionSurgery previously at the same levelsSeverity of disc and facet deg⁡ enerationSeverity of neural space stenosisSeverity of deformity (listhesis,scoliosis,kyphosis)〉+β4〈SurgicalTreatment|DeviceSurgeonInstitutionType,approach,and levels orInvasiveness of surgery〉
 MathType@MTEF@5@5@+=feaafiart1ev1aaatCvAUfKttLearuWrP9MDH5MBPbIqV92AaeXatLxBI9gBaebbnrfifHhDYfgasaacH8akY=wiFfYdH8Gipec8Eeeu0xXdbba9frFj0=OqFfea0dXdd9vqai=hGuQ8kuc9pgc9s8qqaq=dirpe0xb9q8qiLsFr0=vr0=vr0dc8meaabaqaciaacaGaaeqabaqabeGadaaakqaabeqaamaaamaabaqbaeaabiqaaaqaaiabdgeabjabdsgaKjabdAha2jabdwgaLjabdkhaYjabdohaZjabdwgaLbqaaiabd+eapjabdogaJjabdogaJjabdwha1jabdkhaYjabdkhaYjabdwgaLjabd6gaUjabdogaJjabdwgaLbaadaabbaqaauaabaqaceaaaeaacqWGmbatcqWGPbqAcqWGRbWAcqWGLbqzcqWGSbaBcqWGPbqAcqWGObaAcqWGVbWBcqWGVbWBcqWGKbazcqGGSaalcqWGVbWBcqWGYbGCaeaacqWGdbWqcqWGVbWBcqWGUbGBcqWGZbWCcqWGLbqzcqWGXbqCcqWG1bqDcqWGLbqzcqWGUbGBcqWGJbWycqWGLbqzcqWGZbWCaaaacaGLhWoaaiaawMYicaGLQmcacqGH9aqpiiGacqWFYoGydaWgaaWcbaGaeGymaedabeaakmaaamaabaqbaeqabiqaaaqaaiabdcfaqjabdggaHjabdsha0jabdMgaPjabdwgaLjabd6gaUjabdsha0bqaaiabdAeagjabdggaHjabdogaJjabdsha0jabd+gaVjabdkhaYjabdohaZbaadaabbaqaauaabaqadeaaaeaacqWGbbqqcqWGNbWzcqWGLbqzaeaacqWGhbWrcqWGLbqzcqWGUbGBcqWGKbazcqWGLbqzcqWGYbGCaeaacqWGsbGucqWGHbqycqWGJbWycqWGLbqzcqGGVaWlcqWGLbqzcqWG0baDcqWGObaAcqWGUbGBcqWGPbqAcqWGJbWycqWGPbqAcqWG0baDcqWG5bqEaaaacaGLhWoaaiaawMYicaGLQmcacqGHRaWkcqWFYoGydaWgaaWcbaGaeGOmaidabeaakmaaamaabaqbaeaabmqaaaqaaiabdoeadjabd+gaVjabdwgaLjabdIha4jabdMgaPjabdohaZjabdsha0jabdMgaPjabd6gaUjabdEgaNbqaaiabdseaejabdMgaPjabdohaZjabdwgaLjabdggaHjabdohaZjabdwgaLbqaaiabdkeacjabdwha1jabdkhaYjabdsgaKjabdwgaLjabd6gaUbaadaabbaqaauaabaqaeeaaaaqaaiabdkeacjabd+gaVjabdsgaKjabdMha5jabd2eanjabdggaHjabdohaZjabdohaZjabdMeajjabd6gaUjabdsgaKjabdwgaLjabdIha4bqaaiabd2eanjabdwgaLjabdsgaKjabdMgaPjabdogaJjabdggaHjabdYgaSjaaykW7cqWGJbWycqWGVbWBcqWGTbqBcqWGVbWBcqWGYbGCcqWGIbGycqWGPbqAcqWGKbazcqWGPbqAcqWG0baDcqWG5bqEcaaMc8Uaem4Ba8MaemOCaihabaGaemyqaeKaemOBa4MaemyzauMaem4CamNaemiDaqNaemiAaGMaemyzauMaem4CamNaemyAaKMaemyyaeMaaGPaVlabdEgaNjabdkhaYjabdggaHjabdsgaKjabdwgaLbqaaiabdofatjabd2gaTjabd+gaVjabdUgaRjabdMgaPjabd6gaUjabdEgaNjaaykW7cqWGZbWCcqWG0baDcqWGHbqycqWG0baDcqWG1bqDcqWGZbWCaaaacaGLhWoaaiaawMYicaGLQmcaaeaacqGHRaWkcqWFYoGydaWgaaWcbaGaeG4mamdabeaakmaaamaabaqbaeqabmqaaaqaaiabdofatjabdchaWjabdMgaPjabd6gaUjabdggaHjabdYgaSbqaaiabdseaejabdMgaPjabdohaZjabdwgaLjabdggaHjabdohaZjabdwgaLbqaaiabdofatjabdwgaLjabdAha2jabdwgaLjabdkhaYjabdMgaPjabdsha0jabdMha5baadaabbaqaauaabaqafeaaaaqaaiabd6eaojabdwgaLjabdwha1jabdkhaYjabd+gaVjaaykW7cyGGSbaBcqGGVbWBcqGGNbWzcqWGPbqAcqWGJbWycqWGHbqycqWGSbaBcaaMc8UaemOzayMaemyDauNaemOBa4Maem4yamMaemiDaqNaemyAaKMaem4Ba8MaemOBa4gabaGaem4uamLaemyDauNaemOCaiNaem4zaCMaemyzauMaemOCaiNaemyEaKNaaGPaVlabdchaWjabdkhaYjabdwgaLjabdAha2jabdMgaPjabd+gaVjabdwha1jabdohaZjabdYgaSjabdMha5jaaykW7cqWGHbqycqWG0baDcaaMc8UaemiDaqNaemiAaGMaemyzauMaaGPaVlabdohaZjabdggaHjabd2gaTjabdwgaLjaaykW7cqWGSbaBcqWGLbqzcqWG2bGDcqWGLbqzcqWGSbaBcqWGZbWCaeaacqWGtbWucqWGLbqzcqWG2bGDcqWGLbqzcqWGYbGCcqWGPbqAcqWG0baDcqWG5bqEcaaMc8Uaem4Ba8MaemOzayMaaGPaVlabdsgaKjabdMgaPjabdohaZjabdogaJjaaykW7cqWGHbqycqWGUbGBcqWGKbazcaaMc8UaemOzayMaemyyaeMaem4yamMaemyzauMaemiDaqNaaGPaVlGbcsgaKjabcwgaLjabcEgaNjaaykW7cqWGLbqzcqWGUbGBcqWGLbqzcqWGYbGCcqWGHbqycqWG0baDcqWGPbqAcqWGVbWBcqWGUbGBaeaacqWGtbWucqWGLbqzcqWG2bGDcqWGLbqzcqWGYbGCcqWGPbqAcqWG0baDcqWG5bqEcaaMc8Uaem4Ba8MaemOzayMaaGPaVlabd6gaUjabdwgaLjabdwha1jabdkhaYjabdggaHjabdYgaSjaaykW7cqWGZbWCcqWGWbaCcqWGHbqycqWGJbWycqWGLbqzcaaMc8Uaem4CamNaemiDaqNaemyzauMaemOBa4Maem4Ba8Maem4CamNaemyAaKMaem4CamhabaGaem4uamLaemyzauMaemODayNaemyzauMaemOCaiNaemyAaKMaemiDaqNaemyEaKNaaGPaVlabd+gaVjabdAgaMjaaykW7cqWGKbazcqWGLbqzcqWGMbGzcqWGVbWBcqWGYbGCcqWGTbqBcqWGPbqAcqWG0baDcqWG5bqEcaaMc8UaeiikaGIaemiBaWMaemyAaKMaem4CamNaemiDaqNaemiAaGMaemyzauMaem4CamNaemyAaKMaem4CamNaeiilaWIaem4CamNaem4yamMaem4Ba8MaemiBaWMaemyAaKMaem4Ba8Maem4CamNaemyAaKMaem4CamNaeiilaWIaem4AaSMaemyEaKNaemiCaaNaemiAaGMaem4Ba8Maem4CamNaemyAaKMaem4CamNaeiykaKcaaaGaay5bSdaacaGLPmIaayPkJaGaey4kaSIae8NSdi2aaSbaaSqaaiabisda0aqabaGcdaaadaqaauaabaqaceaaaeaacqWGtbWucqWG1bqDcqWGYbGCcqWGNbWzcqWGPbqAcqWGJbWycqWGHbqycqWGSbaBaeaacqWGubavcqWGYbGCcqWGLbqzcqWGHbqycqWG0baDcqWGTbqBcqWGLbqzcqWGUbGBcqWG0baDaaWaaqqaaeaafaqaaeqbbaaaaeaacqWGebarcqWGLbqzcqWG2bGDcqWGPbqAcqWGJbWycqWGLbqzaeaacqWGtbWucqWG1bqDcqWGYbGCcqWGNbWzcqWGLbqzcqWGVbWBcqWGUbGBaeaacqWGjbqscqWGUbGBcqWGZbWCcqWG0baDcqWGPbqAcqWG0baDcqWG1bqDcqWG0baDcqWGPbqAcqWGVbWBcqWGUbGBaeaacqWGubavcqWG5bqEcqWGWbaCcqWGLbqzcqGGSaalcqWGHbqycqWGWbaCcqWGWbaCcqWGYbGCcqWGVbWBcqWGHbqycqWGJbWycqWGObaAcqGGSaalcqWGHbqycqWGUbGBcqWGKbazcaaMc8UaemiBaWMaemyzauMaemODayNaemyzauMaemiBaWMaem4CamNaaGPaVlabd+gaVjabdkhaYbqaaiabdMeajjabd6gaUjabdAha2jabdggaHjabdohaZjabdMgaPjabdAha2jabdwgaLjabd6gaUjabdwgaLjabdohaZjabdohaZjaaykW7cqWGVbWBcqWGMbGzcaaMc8Uaem4CamNaemyDauNaemOCaiNaem4zaCMaemyzauMaemOCaiNaemyEaKhaaaGaay5bSdaacaGLPmIaayPkJaaaaaa@C3CE@

Multiple regression methods such as logistic regression can estimate independent effects of each variable on the likelihood of particular adverse events.

We are evaluating the feasibility and utility of this conceptual model for measuring the safety of different types of lumbar spine surgery. The initial goals of this project are:

(1) to identify the frequency, nature, and severity of adverse occurrences associated with lumbar spine surgery;

(2) to quantify the severity of lumbar degenerative changes;

(3) to quantify the invasiveness of the surgical procedure.

Longer term goals are:

(4) to measure the consequences of adverse events on pain and patient-reported health status two years after surgery; and

(5) to combine these new measures of disease severity and surgical invasiveness with established medical co-morbidity measures in predictive models of adverse events.

In this report, using data from the initial six months of the study, we describe the methods and the preliminary results for the first three goals.

## Methods

### Definitions

We define an adverse occurrence as any medical event in the course of a patient's treatment that has the potential for causing harm to the patient. We selected the term "adverse occurrence" to avoid the connotation of blame often associated with the term "complication." We reserve the term "adverse event" for the subset of adverse occurrences where the patient experiences harm or requires additional monitoring or intervention [20].

### Study design

This report describes research conducted to develop analytical tools for a prospective cohort study of adverse occurrences in lumbar spine surgery. The inclusion and exclusion criteria for the lumbar study are listed in Table 1. The University of Washington (UW) institutional review board approved the study. For this report, we relied on data collected during first six months of that study.

### Outcomes

The primary outcome is a discrete variable that indicates the presence of an adverse occurrence (1 = yes, 0 = no). In the future, we will measure the sensitivity of the safety assessment to different thresholds of adverse occurrence type, etiology, severity, and preventability. In addition to evaluating the association of adverse occurrences with patient, disease, and treatment factors, we will also examine their effect on hospital stay duration, re-admission, re-operation, and patient-reported health status at two years following surgery. We hypothesize that some complications that appear to resolve with treatment post-operatively (e.g., wound infection, cerebrospinal fluid leak) may have lasting effects on pain and function. We are measuring back and leg pain using numerical ratings of intensity and bothersomeness [21-23] and health status by the Short Form-36 [24-26]. We are also measuring pain medication use, work status, and patient satisfaction.

### Ascertaining adverse occurrences

We created *a priori* definitions and ascertainment criteria for 176 adverse occurrences. One orthopedic surgeon and two neurosurgeons specializing in spinal surgery reviewed a list of spine surgery complications [27], eliminated redundancy, and developed explicit definitions for 70 adverse occurrences. Two hospitalists with experience studying surgical complications provided operational definitions for 56 other adverse occurrences [28]. Anesthesiologists experienced in studying anesthetic adverse occurrences provided definitions for 30 peri-operative anesthetic events [29]. With input from operating room nurses, technicians, and managers, we developed criteria for 20 adverse process-of-surgical care issues (e.g., lack of appropriate equipment, implants, documentation, or diagnostic studies). The final list of adverse occurrences and their definitions are provided in the Appendix [see Additional file 1].

In addition to prospective, daily, rigorous medical record review by research staff, we established six other mechanisms for surgeons, residents, fellows, and other team members to independently and voluntarily report adverse occurrences: (1) confidential forms in the operating rooms, inpatient areas, and outpatient clinics with secured collection-boxes; (2) dedicated telephone lines at each hospital; (3) privacy-protected email; (4) weekly spine clinical conferences; (5) daily inpatient rounds; and (6) outpatient clinics [30]. Occurrences from the last three sources were recorded by a designated nurse or physician assistant. We tracked all the modes through which each occurrence was identified.

### Categorizing adverse occurrences

Adverse events in spine surgery are often arbitrarily reported as "device-related," "major," or "preventable." These judgments are not always straightforward, and they profoundly influence interpretation of safety data. Comparisons are difficult unless the terms are applied consistently. We, therefore, used four reviewers to evaluate the consistency of assigning etiology, severity, and preventability to adverse occurrences.

Reviewers were selected from different backgrounds to allow broad clinical perspective. They included a spine fellowship-trained orthopedic surgeon with 7 years of experience, a spine fellowship-trained neurosurgeon with more than 5 years experience, a neurosurgeon with more than 25 years of experience, and an anesthesiologist with more than 5 years of experience. Reviewers individually classified adverse occurrences using pre-established operational definitions [see Additional file 1] and categorization schemes (Tables 2, 3, and 4) and then discussed them as a group in three one-hour training sessions. Subsequently, the four reviewers independently coded adverse occurrences recorded during the first six months of the study.

The reviewers were provided a brief narrative describing each adverse occurrence and the patient's history, surgery, and other information available at discharge. Reviewers were asked to confirm that the reported event met the pre-defined ascertainment criteria and to judge the event's causes, preventability, and severity. Reviewers selected contributing etiological factors from a list of 15 types of errors developed for the Harvard Medical Practice Study and three additional factors for no error (Table 2) [31,32]. Reviewers could select multiple factors, but identified a dominant or most important factor. Reviewers coded preventability as clearly unpreventable, potentially preventable, or clearly preventable [31,32]. For severity coding, we provided the reviewers the adverse event severity categorizing scheme based on the Sentinel Event Reporting Policy required by the Joint Commission on Accreditation of Healthcare Organizations (JCAHO) (Table 3) [33]. By design, this scheme does not distinguish quality of care concerns from patient outcomes, or real effects from potential effects, requiring institutions to define "sentinel event" specifically for their own purposes with "latitude in setting more specific parameters to define 'unexpected,' 'serious,' and 'the risk thereof"' [33]. To measure the impact of adverse occurrences independent of quality of care, with separation of potential risk and actual effect, we developed an "Adverse Occurrence Severity Score" similar to the Index for Categorizing Medication Errors developed by the National Coordinating Council for Medication Error Reporting and Prevention (NCC MERP)(Table 4) [34]. For each adverse occurrence, each reviewer identified the most important factor for etiology, rated preventability, and provided both a JCAHO severity rating and an Adverse Occurrence Severity Score.

### Measuring medical comorbidity

Risk evaluation is crucial to predicting surgical outcomes, but the specific methods most appropriate for spine surgery are unclear. We therefore collected medical comorbidity information using multiple methods. Patients completed a medical history questionnaire to allow calculation of a Charlson comorbidity score [35-37]. We also reviewed medical records to identify presence of 32 medical conditions [38] We additionally recorded the American Society of Anesthesiologists (ASA) grade for anesthetic risk [39] and each patient's height, weight, and tobacco, alcohol, and drug use.

### Measuring disease severity

Lumbar degeneration (spondylosis) is a broad category with varying degrees of severity, and surgical procedures to treat it are individualized to address various aspects of this condition. Technical difficulty of the surgical procedure, and the associated risk of adverse occurrences, may be affected by the anatomical changes, such as the severity of spinal stenosis or the presence and severity of concurrent spondylolisthesis and scoliosis. Also, because patients with more severe and complex spinal disease may seek out particular providers and hospitals, it is important to control for disease severity when comparing adverse occurrences in different settings. We desired a measure of severity of lumbar degeneration to use in predicting the probability of an adverse occurrence.

Using literature review and expert opinions, we developed a severity score using 9 characteristics of degeneration measurable on imaging studies: (1) intervertebral disc signal intensity on magnetic resonance (MR) images [40], (2) intervertebral disc height loss on radiographs or MR images [41], (3) osteophyte formation on radiographs [42,43], (4) disc herniation [44], (5) spinal stenosis [45], (6) spondylolisthesis [46,47], (7) instability on flexion-extension lateral radiographs [48,49], (8) scoliosis [50,51], and (9) kyphosis [52]. We developed definitions for grading severity of each characteristic at each motion segment (Table 5). We also defined a composite "Degenerative Disease Severity Score" as the sum of the scores for each of the 9 imaging dimensions.

To test the reliability of this disease severity scoring method, two observers scored 10 imaging studies of patients showing a broad range of degenerative lumbar spine changes. Image panels showed lumbar spine anterior-posterior and lateral radiographs, lateral flexion and extension views, and sagittal views on MR images. To show the neural tissue space, the panels included an axial image of the spinal canal, sagittal view of the right foramen, and sagittal view of the left foramen for each lumbar level. Each observer rated the 10 cases at two times, approximately 3 weeks apart, identifying a score for each case on all 9 imaging dimensions.

### Measuring surgery invasiveness

Surgical complexity influences risk of adverse occurrences. When comparing different surgeons, hospitals, or devices, the extent and nature of the spinal surgery may be a confounding factor. To control for variations in spinal procedures, we developed a quantitative index to rate the invasiveness of surgery.

We based the index on three fundamental elements of spinal procedures: decompression, fusion, and instrumentation of individual vertebrae. Combinations of these three elements on different vertebrae, when combined with surgical approach (anterior or posterior), can be useful in describing many spinal operations. Each operated vertebra can be assigned a score of 0 to 6, based on how many of six procedural elements were performed at that level: anterior decompression, anterior fusion, anterior instrumentation, posterior decompression, posterior fusion, and posterior instrumentation.

We scored the six constituent procedure components using the following definitions:

(1) Anterior decompression: 1 unit for each vertebra requiring partial or complete excision of the vertebral body or the disc caudal to that vertebra.

(2) Anterior fusion: 1 unit for each vertebra that has graft material attached to or replacing that vertebral body.

(3) Anterior instrumentation: 1 unit for each vertebral body that has screws, plate, cage, or structural graft attached to its vertebral body or replacing its vertebral body.

(4) Posterior decompression: 1 unit for each vertebra requiring laminectomy or foraminotomy at the foramen caudal to its pedicle and/or discectomy at the disc caudal to that vertebral body.

(5) Posterior fusion: 1 unit for each vertebra that has graft material on its lamina, facets, or transverse processes.

(6) Posterior instrumentation: 1 unit for each vertebra that has screws, hooks, or wires attached to its pedicles, facets, lamina, or transverse processes.

**Figure 2:**
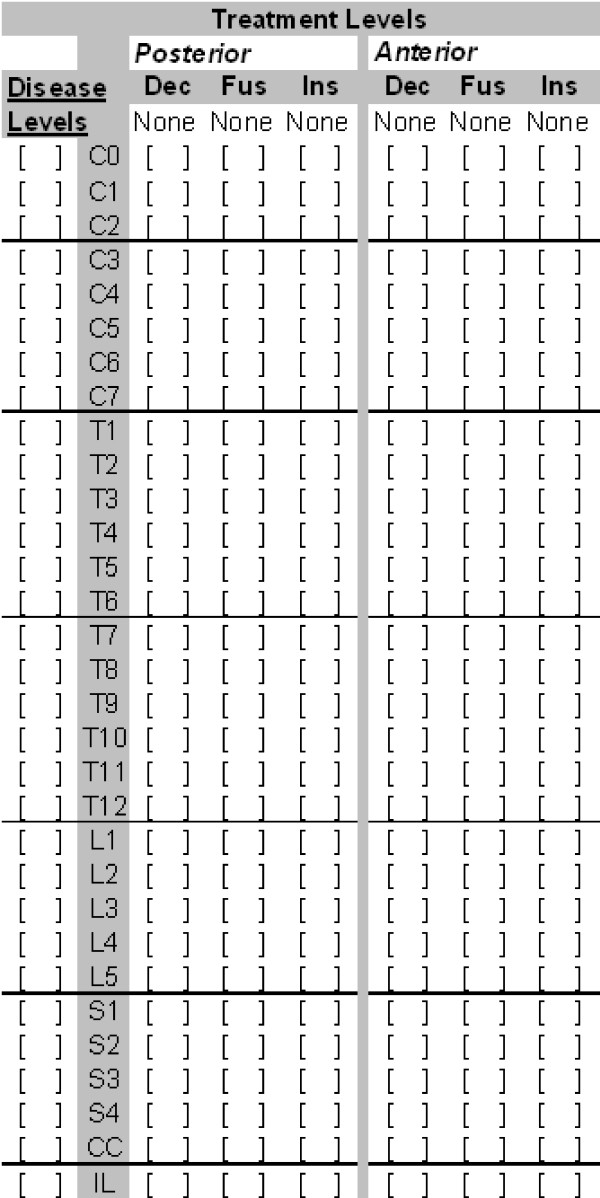
**Graphical Grid for Coding Surgical Procedures. **Graphical grid used to code components of the surgical procedure. Each vertebral level is designated by a row. The columns identify the possible surgical procedures performed at each level: posterior decompression, posterior fusion, posterior instrumentation, anterior decompression, anterior fusion and anterior instrumentation.

Each of the six procedure elements can thus be assigned an integer value corresponding to the number of vertebrae on which that procedural component was performed. We also defined a composite "Spine Surgery Invasiveness Index" as the sum of the six procedural element scores for a given surgery. We developed a graphical grid for coding each surgery (Figure 2).

A surgeon-investigator or a trained research assistant completed the surgical procedure grid based on the treating surgeon's operative report. To determine if this grid method could be reliably used in routine clinical documentation, we made available a medical record form to allow surgeons to record the spinal procedure using the grid format in their immediate hand-written brief operative note. Using the treating surgeon's dictated operation report as the reference, we assessed the reliability of invasiveness coding by comparing the surgeons with the two researchers for fifty consecutive cases.

### Data analysis

We used the kappa statistic to assess agreement between reviewers, using weighted kappa for ranked scales (preventability and JCAHO severity scores) [53,54]. We report kappa values for each pair of observers. Calculations were made using STATA version 8 (College Station, Texas). For evaluating etiology code agreement across four reviewers, we calculated the kappa statistic using the "kap" command in STATA where each observation is assumed to be a subject, the number of raters is fixed (4 raters), and more than two outcomes are possible (18 etiology codes). We set a goal of >0.60 as desirable kappa value for designating agreement as "substantial" or better according to the following published scale [55]:

below 0.0 Poor

0.00-0.20 Slight

0.21-0.40 Fair

0.41-0.60 Moderate

0.61-0.80 Substantial

0.81-1.00 Almost perfect

We assessed agreement on continuous measures (Adverse Occurrence Severity Score, Degenerative Disease Severity Score, and Spine Surgery Invasiveness Index) using intra-class correlation methods using a SAS procedure (SAS Institute, Cary, NC) [56]. We selected the intra-class correlation coefficient (ICC) appropriate for a random sample of reviewers, selected from a larger population, where each reviewer rates each target. We set the significance level (alpha) at 0.05 to calculate 95% confidence intervals (CI).

## Results

### Sample

Between January 1, 2003 and July 1, 2003, 350 patients had lumbar surgical procedures performed at the two participating institutions. Among these, 210 consented for enrollment in the study and 11 declined participation. Patients were offered enrollment only in clinics staffed by a research coordinator, and because of limited resources, only the busiest spine clinics were staffed by research coordinators. Target enrollment for the lumbar spine surgery study is 1000 patients.

### Ascertaining adverse occurrences

During the initial six months of this study, we recorded 172 adverse occurrences for patients undergoing lumbar surgery for degenerative disease. Rigorous daily medical record review identified 92.6% of the total number of adverse occurrences and voluntary reports identified 38.5%; 31.1% of adverse occurrences were identified by both voluntary reports and medical records. Surgeons reported 18.3% of the total number of adverse occurrences ascertained; the inpatient team reported 23.1%, and 6.1% of the total number of adverse occurrences were reviewed or discussed in clinical care conferences, such as morbidity and mortality conferences. Most adverse occurrences were identified only in medical records, such as progress notes, laboratory reports, imaging reports, operation reports, and discharge summaries (61.5%). Surgeons were the sole source for 3.2% and inpatient team members (nurse practitioners, residents, and fellows) were the only source for 4.2%.

### Categorizing adverse occurrences

After classifying some adverse occurrences during the initial training sessions, the four reviewers independently coded the remaining 141 occurrences in 53 patients (Tables 6 and 7). Agreement was substantial for four of the 18 categories of error examined: technical error, failure in communication, systems failure, and no error (Table 8). Agreement across all four reviewers was fair when combined across all 18 error categories, and moderate (using weighted kappa) for preventability and JCAHO severity (Table 9). Numerical severity ratings using the Adverse Occurrence Severity Score showed substantial inter-rater agreement (ICC = 0.74, 95% CI = 0.68 – 0.79).

### Measuring disease severity

**Figure 3:**
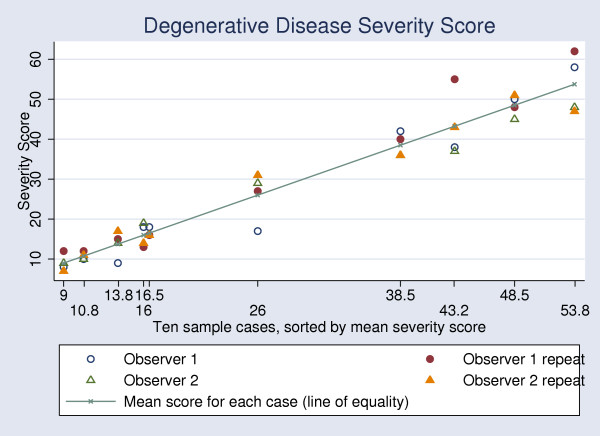
**Degenerative Disease Severity Score. **The degenerative disease severity score assigned by two observers for 10 sample cases. Score by Observer 1 highly correlates with the score given by Observer 2 and with repeat scores for each observer.

Overall (mean) agreement for disease severity dimensions was moderate across observers and substantial within observers (Table 10). Inter-observer agreement was lowest for herniation and instability and strongest for degeneration. There was excellent agreement for the Degenerative Disease Severity Score (ICC = 0.98, 95%CI = 0.96 – 0.99) (Figure 3).

### Measuring surgery invasiveness

**Figure 4:**
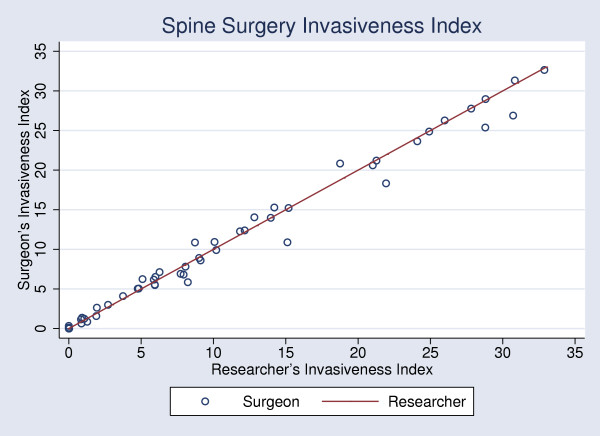
**Spine Surgery Invasiveness Index. **Spine Surgery Invasiveness Index assigned by the treating surgeon and two researchers for 50 consecutive operations.

Inter-researcher agreement was almost perfect for the Invasiveness Index and for its six constituent elements (Table 11). Surgeons completed the grid operative report form as part of their medical record documentation in only 53% of the cases. Agreement between the surgeons and the researchers was very high on the forms completed (Table 10) (Figure 4).

## Discussion

Adverse occurrences are unwanted but common, often carrying burdens of blame, guilt, or fear of sanctions [57,58]. Terms such as complication, adverse event, and medical error exacerbate the punitive atmosphere surrounding undesirable outcomes, particularly when these events are related to surgical procedures [59,60]. As a result, despite a century-old tradition among surgeons to focus intensely on complications in regular morbidity and mortality conferences [61], discussions of adverse occurrences in the surgical literature are frequently dismissive or defensive, leaving lessons buried under quality assurance protections [62]. Sanitized or closed quality-of-care discussions prevent systematic review of experience across institutions or cumulative experience over time, restricting knowledge that may prevent future occurrences [63]. Mistakes get repeated. Patient safety suffers.

Approaches to measuring the safety of spine surgery are not well-developed. We undertook preliminary evaluations to help define a protocol to monitor adverse occurrences associated with spine surgery. We chose a design engineering perspective to create a conceptual framework with desirable components and specifications, including multi-modal, standardized, comprehensive surveillance of outcomes and detailed measurement of risk-adjustment factors. Establishing multiple methods to track 176 adverse occurrences requires extensive resources and is not practical for routine clinical surveillance. Identifying the most common or most severe of these events may help to select a smaller set of indicator events. Since many adverse occurrences tended to cluster in cascades, understanding associations among occurrences may allow selection of a shorter list of critical surveillance items. Quantifying disease severity on imaging studies and surgical invasiveness from medical records requires additional extensive resources. While such a complex and bulky system can be implemented in rigorous regulatory approval studies of new devices or other well-funded trials, widespread acceptance and application may require selecting subsets of risk factors and adverse outcomes that directly relate to specific patient safety concerns, or choosing those parameters in this framework that can be ascertained reliably from brief medical record reviews or administrative data alone.

Comprehensive surveillance of all adverse occurrences is difficult, if not impossible. Tracking surgical complications may be particularly troublesome because of issues relating to responsibility and liability surrounding invasive interventions. Although the true number of adverse occurrences cannot be determined, our experience confirms that complementary surveillance methods provide more complete assessment [64]. Our multi-modal attempt for capturing adverse occurrences showed that self-report by surgeons was not sufficient for identifying most adverse occurrences, and neither was reliance on voluntary reports by the spine team conducting daily ward rounds. Contrary to experience reported for some settings [30], in our study even designated professionals integrated into the daily team rounds were not sufficient to discern most adverse occurrences, perhaps because these personnel were not consistently aware of intra-operative occurrences, near-miss occurrences, or occurrences only observed by consulting services. Also, surgical team members may not have completely trusted the study goals during the early study period reported here. Hopefully, voluntary reporting can improve as team members develop greater awareness of reporting methods, more certainty that prevention through learning is the sole motive for surveillance, and in time, cultivate a culture of safety that encourages openness.

Categorizing adverse occurrences is problematic. Reviewers agreed in their discrimination of error from no error, and they consistently identified errors related to technical, communication, or systems failures. They were also able to reliably assign severity ratings to adverse outcomes using a scale that separated actual from potential effects. Reviewers, however, had difficulty determining preventability of adverse occurrences and assessing severity using a classification based on the JCAHO Sentinel Event Policy. Adverse occurrences are products of complex patient and treatment factors, often occurring in cascades where it is difficult to isolate the causes and effects of individual events. Reviewer agreement may be limited in part due to lack of detailed information. Also, some consequences may not be apparent at the time of hospital discharge, when ratings were assigned. Agreement among reviewers may improve with more experience, with provision of more detailed narratives, or with development of simpler coding scales.

Initial assessment of severity scoring for degenerative changes in the lumbar spine is promising. Two orthopedic surgeons showed good agreement in distinguishing patients with mild degeneration from those with severe degenerative changes. More work is needed to assess generalizability and to describe how different aspects of degeneration may be related to presenting symptoms and functional impairment. Such research may allow hierarchical ranking of broad diagnostic categories within lumbar spondylosis or permit weighting of different components of degeneration.

Surgical procedures on the spine can be quantitatively ranked for invasiveness. Although surgeons were only able to provide this information routinely in just over half the cases, when the information was provided, it was reliable. Compliance may improve with time, encouragement, or proof of the value of such coding. Validation of this ranking system by comparison to other indicators of invasiveness, such as duration of surgery or blood loss, may help better assess utility of the ranking system and add meaning to the relative invasiveness of various procedural elements.

Our study only included the busiest spine centers within our network. This choice may have introduced bias. Surgical volume may influence both the frequency and the reporting of adverse occurrences. Busier centers and surgeons may have lower rates of some occurrences and higher rates of others. Incorporating additional tasks of surveillance and reporting into routine care processes may be more difficult in busy, high-volume settings. Some of these concerns could be addressed by limiting surveillance to only a select few adverse occurrences that are routinely recorded in operation reports and hospital discharge summaries.

Our study placed emphasis on explicitly recording absence of an adverse occurrence when none occurred. Lack of occurrence of a particular complication with a particular procedure is important information. The efficiency of surveillance of what occurred cannot be judged without explicit data on what did not occur. No report does not equal no occurrence. To be meaningful, adverse occurrence reports should specify what was monitored, how often it occurred, and how often it did not occur.

We hope that sharing this protocol development will stimulate discussion of these methodological issues and push the field towards greater standardization in reporting and comparing adverse occurrence rates for devices, techniques, and healthcare providers. Although our focus is lumbar surgery for degenerative disease, the methods described may be applicable also to surgery in other regions of the spine. The analytic approach described may also have relevance for efficacy level evaluation of current and new procedures. Individual hospital and provider level analyses may be useful for effectiveness research and quality improvement.

## Conclusion

Approach to measuring the safety of spine surgery can be standardized. Scales for rating the impact of adverse occurrences, severity of lumbar spine degeneration, and invasiveness of spine surgery have acceptable reproducibility. Reviewers frequently disagree on causes of adverse occurrences.

## Competing interests

Support of spine-related research at the University of Washington (UW) includes a gift of an endowed chair established in 1999 by support from Surgical Dynamics, a past manufacturer of spinal implants, to conduct outcomes research in spine surgery. The UW Department of Orthopedics has also received gifts of endowed chairs from Synthes (Paoli, PA) in 2003 and Depuy Spine (Rayhnam. MA) in 2005, current manufacturers of spinal implants. Synthes and Depuy also provide spine fellowship support at UW. In addition, Synthes has established a Spine End-Results Research (SERR) Fund at UW for conducting safety and outcomes research on spine surgery patients. The principal investigator for this fund is a faculty member in the orthopedics department and the fund is managed through the Grant and Contract Services Office of the University of Washington. The sponsors of the endowments and the research fund have no control over design, conduct, data, analysis, review, reporting, or interpretation of clinical research conducted with the funds. SM and the University of Washington also hold two patents on surgical drills. These patents are licensed by Synthes. SM and the University of Washington do not conduct research to evaluate use of these surgical drills in patients.

## Authors' contributions

SM and RD designed the study. SM, RD, PH, and JT developed the research proposal. SM, LL, and RG implemented the research methods and assisted with data collection. SM supervised data collection. SM, RD, and PH designed data analyses. SM conducted the analyses. All authors reviewed, edited, and approved the manuscript.

## Pre-publication history

The pre-publication history for this paper can be accessed here:



## Supplementary Material

Additional File 1 An Appendix is provided as an additional file in Microsoft^® ^Office Word 2003 format. It contains operational definitions established *a priori *for adverse occurrence surveillance in this study. Definitions for occurrences related to escalation of care and airway management (ec00 to mazz) are adapted from *Posner et al (Posner KL, Freund PR. Trends in quality of anesthesia care associated with changing staffing patterns, productivity, and concurrency of case supervision in a teaching hospital. Anesthesiology 1999;91(3):839-47)*. Medical occurrences (mc00 to muzz) are adapted from Reilly et al *(Reilly DF, McNeely MJ, Doerner D, et al. Self-reported exercise tolerance and the risk of serious perioperative complications. Arch Intern Med 1999;159(18):2185-92) *with additional details obtained from Harrison's textbook of Medicine *(Fauci AS, Braunwald E, Isselbacher KJ, et al., eds. Harrison's Principles of Internal Medicine 14th Edition. Philadelphia: McGraw-Hill; 1998)*. Remaining definitions were developed by the study team with reference to the published literature when available. Additional information, such as itemized criteria for ascertainment and related published references, are available from our study team. Click here for file

## Figures and Tables

**Table 1:** Inclusion and exclusion criteria.^1^

Inclusion criteria:
1. Age greater than 18 years (to allow informed consent).
2. Diagnosis is lumbar degenerative disease (disc degeneration, disc herniation, spinal stenosis, spondylolisthesis, or degenerative scoliosis).
3. Surgery involves at least one lumbar vertebra.
4. Surgery at Harborview Medical Center or University of Washington Medical Center.
Exclusion criteria:
1. Inflammatory spondyloarthropathy.
2. Spinal malignancy or infection.
3. Pregnancy.
4. No telephone contact, or planning to move within a year.
5. Unable to complete study questionnaires or follow-up telephone interviews in English.

^1^Concurrent with the initiation of this study, we established a spine registry to track safety and outcomes of all spine surgery procedures at the University of Washington (the Spine End Results Registry). Research coordinators attempt to offer most patients scheduled for spine surgery at the University of Washington the opportunity to enroll in the registry, but because of limited staff, only the busiest spine clinics are staffed with research coordinators. The criteria listed here specify the subset of registry patients selected for studying lumbar surgery for degenerative disease.

**Table 2:** Harvard Medical Practice Study categories for classifying etiology of adverse events and medical errors, with three added categories for patient factors.

**Code**	**Type of Error**	**Description**
1	Diagnostic	Error in diagnosis or delay in diagnosis
2	Diagnostic	Failure to employ an indicated test
3	Diagnostic	Use of outmoded tests or therapy
4	Diagnostic	Failure to act on the results of monitoring or testing
5	Treatment	Technical error in performance of an operation, procedure, or test
6	Treatment	Error in administering the treatment (including preparation for operation or treatment)
7	Treatment	Error in dose of drug or in the method of use of a drug
8	Treatment	Avoidable delay in treatment or in responding to an abnormal test
9	Treatment	Inappropriate (not indicated) care. Considering the patient's disease, its severity, and comorbidity, the anticipated benefit from the treatment did not significantly exceed the known risk, or a superior alternative was available
10	Preventive	Failure to provide indicated prophylactic treatment
11	Preventive'	Inadequate monitoring or follow-up of treatment
12	System	Failure in communication
13	System	Equipment failure
14	System	Other systems failure
15	Other	Unclassified
*16	No error	Patient disease, expected risk
		
*17	No error	Patient non-compliance
*18	No error	Patient disease, unrelated to spinal surgery

*Additional three categories not included in the Harvard Medical Practice Study. We group these as "no error" simply to distinguish them from the evaluation and treatment associated factors under direct control of the medical care system. Alternatively, these three factors may be considered errors in patient selection.

**Table 3:** Severity rating based on the JCAHO Sentinel Event Policy for adverse events not related to the natural course of the patient's illness or underlying condition.

**Code**	**Description**
0	No quality of care concerns evident.
1	Did not and unlikely to have had an adverse effect.
2	Did not but had the potential to have had an adverse effect.
3	Had an adverse effect but not life threatening.
4	Resulted in loss of major physical function or potentially life threatening.
5	Demonstrated a life threatening situation or resulted in death.

**Table 4:** Adverse Occurrence Severity Score developed to distinguish actual effect from the magnitude of risk associated with adverse occurrences.

**Score**	**Summary**	**Description**
0	No effect, no risk	Adverse occurrence required no intervention, resulted in no adverse consequences, and had no risk of adverse consequences.
1	No effect, minor risk	Adverse occurrence required no intervention, resulted in no adverse consequences, but had the potential to result in minor consequences.
2	No effect, major risk	Adverse occurrence required no intervention, resulted in no adverse consequences, but had the potential to result in major but not life threatening adverse consequences.
3	No effect, risk of death	Adverse occurrence required no intervention, but had the potential to result in a life-threatening situation or death.
4	Minor effect, minor risk	Adverse occurrence required a minor intervention or resulted in minor loss of function, and had the potential to result in only minor adverse consequences.
5	Minor effect, major risk	Adverse occurrence required a minor intervention or resulted in minor loss of function, but had the potential to result in major loss of function, though not life-threatening.
6	Minor effect, risk of death	Adverse occurrence required a minor intervention or resulted in minor loss of function, but had the potential to result in a life-threatening situation or death.
7	Major effect, major risk	Adverse occurrence required extensive intervention such as unexpected re-operation or re-admission, or resulted in major loss of function, but was not life-threatening.
8	Major effect, risk of death	Adverse occurrence required extensive intervention such as unexpected re-operation or re-admission, or resulted in major loss of function, and had the potential to result in a life-threatening situation or death.
9	Life-threatening effect	Adverse occurrence resulted in a life-threatening situation.
10	Death	Adverse occurrence resulted in death.

**Table 5:** Nine subscales for scoring the severity of degenerative changes in the lumbar spine on imaging studies.

**1. Degeneration**: assign a value 0 to 3 at each level		
None	0	
Dark disc on T2 MRI	**1**	
End plate edema on T2 MRI	**2**	
End plate sclerosis	**3**	
		
**2. Height loss**: assign a value 0 to 3 at each level		
None	0	
Yes, < 50%	1	
Yes, > 50%, but not ankylosis	2	
Yes, ankylosis	3	
		
**3. Osteophytes**: assign a value 0 to 3 at each level		
None	0	
Yes, < 2 mm	1	
Yes, > 2 mm but not bridging	2	
Yes, bridging	3	
		
**4. Herniation**: assign a value 0 to 4 at each level		
None	0	
Bulge	1	
Protrusion	2	
Extrusion	3	
Sequestered	4	
		
**5. Stenosis**: assign the total score 0 to 6 for each level		
Right foramen	0 or 1	*Value for stenosis is the sum of these components or 6 if there is complete block*.
Left foramen	0 or 1	
Right lateral recess	0 or 1	
Left lateral recess	0 or 1	
Central stenosis	0 or 1	
Complete block	6	
		
**6. Listhesis**: assign a value 0 to 5 for each level		
None, <10%	0	
Grade 1, 10 to 25%	1	
Grade 2, 26 to 50%	2	
Grade 3, 51 to 75%	3	
Grade 4, 76 to 100%	4	
Grade 5, > 100%	5	
		
**7. Instability**: assign an instability score 0 to 3 for each level		
No instability	0	
Mild instability	1	
Moderate instability	2	
Severe instability	3	
		
**8. Scoliosis and 9. Kyphosis**: specify total magnitude 0 to 6 for each patient		
<10 degrees	0	
11 to 19 degrees	1	
20 to 29 degrees	2	
30 to 39 degrees	3	
40 to 49 degrees	4	
50 to 59 degrees	5	
> 60 degrees	6	

**Table 6:** The sources for the pre-defined adverse occurrences coded by all four reviewers independently after the initial training sessions.

**Categories**	**Number**	
**Surgical List**		**56**
*Iatrogenic injury*	*25*	
*Device problems*	*16*	
*Diagnosis problems*	*4*	
*Wound problems*	*11*	
**Medical List**		**70**
*Respiratory events*	*18*	
*Hematological events*	*17*	
*Urological events*	*12*	
*Neurological events*	*8*	
*Cardiac events*	*7*	
*Drug-related events*	*4*	
*Gastrointestinal events*	*2*	
*Vision problems*	*2*	
**Anesthetic List**		**10**
*Escalation of care*	*7*	
*Airway problems*	*3*	
**Management List**		**5**
*Delay in surgery*	*3*	
*Process of care issues*	*2*	

**Total**		**141**

**Table 7:** Clustering among patients of adverse occurrences reviewed independently by all four reviewers.^1^

1	adverse occurrence in	22	patients
2	adverse occurrences in	9	patients
3	adverse occurrences in	7	patients
4	adverse occurrences in	6	patients
5	adverse occurrences in	5	patients
6	adverse occurrences in	1	patient
7	adverse occurrences in	1	patient
9	adverse occurrences in	2	patients
**Total**			
**141**	**adverse occurrences in**	**53**	**patients**

^1 ^Among the patients with an adverse occurrence, more than half (31/53) experienced multiple events. This clustering made cause, effect, and preventability judgments on individual events difficult.

**Table 8:** Etiology categories: Agreement among all four observers for 141 adverse occurrences coded by each reviewer.^1^

**Etiology Code**^2^	**Category**	**Kappa**	**p-value**
**1**	Error in diagnosis	0.52	0.0000
**4**	Failure to act on results	0.33	0.0000
**5**	**Technical error**^3^	**0.66**	**0.0000**
**6**	Error in preparation for operation	0.09	0.0064
**7**	Error in dose or method of use of a drug	0.21	0.0000
**8**	Avoidable delay in treatment	0.15	0.0000
**9**	Inappropriate care	0.06	0.0313
**10**	Failure to provide indicated prophylactic treatment	0.11	0.0007
**11**	Inadequate monitoring or follow-up	0.05	0.0738
**12**	**Failure in communication**^3^	**0.80**	**0.0000**
**13**	Equipment failure	0.33	0.0000
**14**	**Other systems failure**^3^	**0.85**	**0.0000**
**15**	Unclassified error	0.01	0.3453
**16**	Patient disease, expected risk (no error)	0.26	0.0000
**17**	Patient non-compliance (no error)	0.00	0.5206
**18**	Disease unrelated to spine surgery (no error)	0.22	0.0000
**16, 17, or 18**	**No error (16, 17, or 18)**^3^	**0.61**	**0.0000**

----	Combined (for all categories)	0.35	0.0000

^1 ^Kappa statistic calculated using "kap" command in STATA Version 8 (College Station, TX). Each observation is assumed to be a subject, the number of raters is fixed (4 raters), and more than two outcomes are possible (18 etiology codes).

^2 ^None of the 141 adverse occurrences were assigned etiology codes 2 or 3.

^3 ^These categories show substantial or better agreement, with kappa values = 0.61

**Table 9:** Etiology, preventability, and the JCAHO severity ratings: Agreement between pairs of observers for 141 adverse occurrences coded by all four reviewers.

	**O-A^1^**	**O-JN^2^**	**O-SN^3^**	**A-JN^4^**	**A-SN^5^**	**JN-SN^6^**	**Mean^7^**
Etiology, kappa	0.33	0.33	0.33	0.43	0.45	0.28	0.36
Preventability, weighted kappa	0.33	0.60	0.56	0.24	0.43	0.49	0.44
JCAHO Severity, weighted kappa	0.25	0.57	0.34	0.26	0.21	0.31	0.33

^1^Agreement between the orthopedic surgeon reviewer(O) and the anesthesiologist(A).

^2^Agreement between the orthopedic surgeon reviewer(O) and the junior neurosurgeon(JN).

^3^Agreement between the orthopedic surgeon reviewer(O) and the senior neurosurgeon(SN).

^4^Agreement between the anesthesiologist(A) and the junior neurosurgeon(A).

^5^Agreement between the anesthesiologist(A) and the senior neurosurgeon(SN).

^6^Agreement between the junior neurosurgeon(JN) and the senior neurosurgeon(SN).

^7 ^Mean for all six pairs of reviewers.

**Table 10:** Disease Severity Scoring: Agreement between and within observers for 9 imaging disease characteristics for 10 patients. Each observer scored each case initially and then again approximately three weeks later.

**Imaging Dimension**	**Observer 1 vs 2^1^**	**Observer 1^2^**	**Observer 2^3^**
1. Degeneration	0.70	0.72	0.85
2. Height Loss	0.44	0.49	0.63
3. Osteophytes	0.47	0.53	0.67
4. Herniation	0.28	0.61	0.41
5. Stenosis	0.44	0.37	0.56
6. Listhesis	0.54	0.64	0.83
7. Instability	0.38	-0.02	1.00
8. Scoliosis Magnitude	0.51	1.00	0.58
9. Kyphosis Magnitude	0.42	0.62	0.62

**Mean**	**0.45**	**0.58**	**0.67**

^1 ^Kappa value for inter-observer agreement between Observer 1 and Observer 2.

^2 ^Kappa value for intra-observer agreement for Observer 1.

^3 ^Kappa value for intra-observer agreement for Observer 2.

**Table 11:** Surgery Invasiveness Scoring: Inter-rater agreement for procedure invasiveness measurements for 50 consecutive operations coded by the treating surgeon and two researchers.

**Procedure Characteristic**	**Researchers**^1^	**Surgeons**^2^
	***ICC (95%CI)***	***ICC (95%CI)***
Invasiveness Index	0.998 (0.997 to 0.999)	0.995 (0.993 to 0.997)
Anterior decompression	0.995 (0.992 to 0.997)	0.872 (0.814 to 0.920)
Anterior fusion	0.992 (0.988 to 0.995)	0.912 (0.872 to 0.945)
Anterior instrumentation	0.994 (0.991 to 0.996)	0.923 (0.887 to 0.951)
Posterior decompression	1.000	0.992 (0.989 to 0.995)
Posterior fusion	0.999 (0.999 to 1.000)	1.000
Posterior instrumentation	1.000	0.996 (0.994 to 0.997)

^1^Intraclass correlation coefficients for agreement between the two researchers: surgeon-investigator and a trained research assistant.

^2^Intraclass correlation coefficients for agreement between the treating surgeon and the researchers.
