# Supplementary material for: Towards standardized measurement of adverse events in spine surgery: conceptual model and pilot evaluation
Source: BMC Musculoskelet Disord. 2006 Jun 20;7:53. doi: 10.1186/1471-2474-7-53 (PMC1562418; doi:10.1186/1471-2474-7-53)
Supplement: Additional File 1 — An Appendix is provided as an additional file in Microsoft® Office Word 2003 format. It contains operational definitions established a priori for adverse occurrence surveillance in this study. Definitions for occurrences related to escalation of care and airway management (ec00 to mazz) are adapted from Posner et al (Posner KL, Freund PR. Trends in quality of anesthesia care associated with changing staffing patterns, productivity, and concurrency of case supervision in a teaching hospital. Anesthesiology 1999;91(3):839-47). Medical occurrences (mc00 to muzz) are adapted from Reilly et al (Reilly DF, McNeely MJ, Doerner D, et al. Self-reported exercise tolerance and the risk of serious perioperative complications. Arch Intern Med 1999;159(18):2185-92) with additional details obtained from Harrison's textbook of Medicine (Fauci AS, Braunwald E, Isselbacher KJ, et al., eds. Harrison's Principles of Internal Medicine 14th Edition. Philadelphia: McGraw-Hill; 1998). Remaining definitions were developed by the study team with reference to the published literature when available. Additional information, such as itemized criteria for ascertainment and related published references, are available from our study team. [file 1471-2474-7-53-S1.doc]

# Appendix

This appendix contains operational definitions we established *a priori* for adverse occurrence surveillance in our study. Definitions for occurrences related to escalation of care and airway management (ec00 to mazz) were adapted from Posner et al (Posner KL, Freund PR. Trends in quality of anesthesia care associated with changing staffing patterns, productivity, and concurrency of case supervision in a teaching hospital. Anesthesiology 1999;91(3):839-47). Medical occurrences (mc00 to muzz) were adapted from Reilly et al (Reilly DF, McNeely MJ, Doerner D, et al. Self-reported exercise tolerance and the risk of serious perioperative complications. Arch Intern Med 1999;159(18):2185-92). Additional details where necessary were obtained from Harrison’s textbook of Medicine (Fauci AS, Braunwald E, Isselbacher KJ, et al., eds. Harrison's Principles of Internal Medicine 14th Edition. Philadelphia: McGraw-Hill; 1998). Remaining definitions were developed by the study team with reference to the published literature when available. Additional information, such as criteria for ascertainment and specific references, are available from our study team.

*Code:* **aa00 General**

*Operational definition:* Category for adverse occurrences not otherwise specified as medical events, technical events, wound problems, late events, or process issues, including major complications such as reoperation, CPR, and death.

*Code:* **aa01 No unexpected occurrence**

*Operational definition:* There were no unexpected occurrences recognized on the date of this entry. If recorded on the surgery information form, this designates that no adverse occurrences were recognized during surgery. This designation on the hospital discharge form designates no adverse occurrences during hospitalization. On the follow-up form, this designation indicates no adverse occurrences recognized or being treated on that date.

*Code:* **aa02 Death**

*Operational definition:* Death is the permanent cessation of all vital bodily functions:total cerebral function; spontaneous respiratory function; and spontaneous circulatory function. Death may be recorded in the medical record or reported by a family member or other sources.

*Code:* **aa03 CPR**

*Operational definition:* The patient required cardiopulmunary resuscitation: chest compressions and external respirations or mechanical ventilation.

*Code:* **aa04 Identification of patient or incision site**

*Operational definition:* The patient who started undergoing evaluation or treatment was not the planned patient, or the incision was made at a spinal region or side different from that planned.

*Code:* **aa05 Surgery aborted after incision**

*Operational definition:* The procedure was discontinued temporarily or definitively after making the initial incision; or the wound was packed emergently or provisionally closed with or without sutures. This includes procedures that may have resumed after stabilization of the patient on the same day under the same anesthetic.

*Code:* **aa06 Reoperation, unexpected**

*Operational definition:* The patient required a subsequent unplanned surgical procedure at any time after the index spine procedure, and this secondary procedure was related to or caused by the index spine procedure.

*Code:* **aa07 ICU transfer, unexpected (ICU,RICU,tele)**

*Operational definition:* The patient required an unplanned transfer from the Orthopaedics or Neurosurgical Units to an Intensive Care Unit or unplanned ICU admission was necessary after surgery.

*Code:* **aa08 Ventilator, unexpected**

*Operational definition:* The patient was unexpectedly placed on a mechanical ventilator.

*Code:* **aa09 Code blue/199 activitaion for any reason**

*Operational definition:* Medical center personnel called a code 199 any time from the admission of the patient until discharge.

*Code:* **dl00 Delay**

*Operational definition:* Category for adverse occurrences involving a delay in care due to organizational processes.

*Code:* **dl01 delay,disposition/placement for hosp d/c**

*Operational definition:* The patient cold not be discharged for more than 48 hours beyond the time the patient was medically ready, and the delay was due to difficulty in obtaining placement in a care facility.

*Code:* **dl02 delay, patient condition ______d**

*Operational definition:* The procedure was delayed more than 1 day due to an inadequate workup or change in the patient's medical condition.

*Code:* **dl03 delay, imaging availability __ ___d**

*Operational definition:* The procedure was delayed (more than 1 day for an inpatient or more than 1 hour in the operating room) because lack of imaging availability.

*Code:* **dl04 delay, SEP availability ______d**

*Operational definition:* The procedure was delayed (more than 1 day for an inpatient or more than 1 hour in the operating room) due to SSEP unavailability.

*Code:* **dl05 delay, OR turnover ______h**

*Operational definition:* The procedure was delayed (more than 1 day for an inpatient or more than 1 hour in the operating room) due to lack of OR availability.

*Code:* **dl06 delay, implant availability ______h**

*Operational definition:* The procedure was delayed (more than 1 day for an inpatient or more than 1 hour in the operating room) due to lack of a required implant or implant-specific tool.

*Code:* **dl07 delay, surgeon availability ______h**

*Operational definition:* The procedure was delayed (more than 1 day for an inpatient or more than 1 hour in the operating room) due to lack of a required surgeon (or surgeons).

*Code:* **dlzz other delay**

*Operational definition:* Other delay in care that resulted in adverse medical consequences for the patient.

*Code:* **dx00 Diagnosis**

*Operational definition:* Category for adverse occurrences related to the recognition or diagnosis of a medical condition.

*Code:* **dx01 delayed recognition of adverse occurrence**

*Operational definition:* An adverse occurrence was not recognized until the patient developed preventable irreversible medical consequences.

*Code:* **dx02 suboptimal preop evaluation resulting in ao**

*Operational definition:* An adverse event occurred which may have been prevented by more specific preoperative evaluation of the patient, and the adaequate preoperative evaluation would have typically prevented the adverse event.

*Code:* **dx03 delayed diagnosis of neural deficit**

*Operational definition:* A motor or sensory neurologic deficit was present, and earlier diagnosis would have changed management of the patient.

*Code:* **dx04 missed diagnosis of neural deficit**

*Operational definition:* Motor or sensory deficit requiring specific intervention was not diagnosed prior to definitive spinal treatment, surgery, hospital dishcharge, or adverse outcome.

*Code:* **dx05 delayed diagnosis of vertebral injury**

*Operational definition:* A vertebral column injury (fracture, dislocation, ligament failure, disk protrusion) requiring specific treatment was not diagnosed until definitive spinal treatment, surgery, hospital dishcharge, or adverse outcome occurred, and earlier diagnosis would have changed management of the patient.

*Code:* **dx06 missed diagnosis of vertebral injury**

*Operational definition:* A vertebral column injury (fracture, dislocation, ligament failure, disk protrusion) requiring specific treatment was not diagnosed until definitive spinal treatment, surgery, hospital dishcharge, or adverse outcome occurred.

*Code:* **dxzz other diagnosis occurrence**

*Operational definition:* Other diagnosis-related occurrence.

*Code:* **ec00 Escalation of Care**

*Operational definition:* Category for adverse occurrences in which the patient required additional monitoring or intervention than was initially planned or routinely needed.

*Code:* **ec01 change in anesthetic plan**

*Operational definition:* Any change in original plan caused by worsening or unsuspected patient condition, such as increased monitoring.

*Code:* **ec02 change in surgical plan**

*Operational definition:* Any change in original surgical plan: (1) caused by worsening or unsuspected patient condition, such as staging procedure or packing uncontrolled hemorrhage due to unstable hemodynamics; or (2) intra-operative findings or adverse occurrences requiring additional lvevels of decompression, fusion, or instrumentation; or (3) other interventions not initially planned.

*Code:* **ec03 delay in extubation in OR**

*Operational definition:* Delay > 15 minutes for extubation after patient is turned supine and imaging is completed.

*Code:* **ec04 delay in extubation in PACU**

*Operational definition:* Delay > 15 minutes for extubation after arrival to PACU (extrapolation from Overdyk's definition for delay in extubation in OR; does not include planned overnight mechanical ventilation for laryngotracheal edema.)

*Code:* **ec05 extra drugs required during surgery**

*Operational definition:* Any additional drugs required during surgery due to worsening or unsuspected patient condition which were not part of original anesthesia plan. E.g., use of vasopressors / inotropes to support hemodynamics

*Code:* **ec06 extra tests required during surgery**

*Operational definition:* Any additional tests required during surgery due to worsening or unsuspected patient condition which were not part of original anesthesia plan. E.g., troponin and CK-MB lab tests.

*Code:* **ec07 prolonged stay in PACU**

*Operational definition:* Any stay in PACU > 1 hours for medical reasons only. E.g., inadequate oxygenation / ventilation.

*Code:* **ec08 reintubation**

*Operational definition:* The patient was intubated at any time after being extubated following the procedure.

*Code:* **eczz other escalation of care**

*Operational definition:* Other escalation of care.

*Code:* **ma00 Medical: Airway**

*Operational definition:* Category for adverse occurrences related to airway management.

*Code:* **ma01 airway edema**

*Operational definition:* Stridor or laryngeal dyspnea or prolonged inspiration with accessory usage of respiratory muscles or crowing sound with inspiration, or reintubation with evidence of edema on laryngoscopy, or patient remained intubated overnight because of airway edema as evidenced by significant facial swelling and/ or absence of leak when pilot balloon deflated.

*Code:* **ma02 airway trauma**

*Operational definition:* Airway injury to lips, teeth, nose, oropharynx, larynx, trachea or esophagus caused by manipulation of the airway.

*Code:* **ma03 airway obstruction**

*Operational definition:* Increased airway resistance caused by upper airway obstruction in the pharynx, larynx, or large airways, or by obstruction of the endotracheal tube, causing hypoxemia (SpO2 < 90%) or hypercarbia (PaCO2 > 60mm Hg), and requiring an active and specific intervention.

*Code:* **ma04 aspiration**

*Operational definition:* any obvious nonrespiratory secretions suctioned via an endotracheal tube; CXR evidence of new pathology after an incident and/or there were signs of new wheeze or crackels after an episode of regurgitation or vomiting.

*Code:* **ma05 brochospasm(wheezing/nebulizer/steroid)**

*Operational definition:* Any audible wheeze or unexplained increase in airway pressure requiring an active and specific intervention.

*Code:* **ma06 accidental extubation**

*Operational definition:* Unplanned extubation by caregivers or patient.

*Code:* **ma07 difficult intubation**

*Operational definition:* A normally trained anesthesiologist required more than 3 attempts or more than 10 minutes for a successful endotracheal intubation.

*Code:* **ma08 endobronchial intubation**

*Operational definition:* The tip of the endotracheal tube resides in either the left or right mainstem bronchus, as evidenced by hypoxemia (SpO2 < 90%) or lung collapse by CXR.

*Code:* **ma09 esophageal intubation**

*Operational definition:* Esophageal intubation is unintended placement of the breathing tube into the esophagus.

*Code:* **ma10 inability / difficult to ventilate**

*Operational definition:* Inability or difficulty to exchange adequate tidal volumes to prevent hypercarbia (PaCO2 > 60 mm Hg) or hypoxemia (SpO2 < 90%)

*Code:* **ma11 inadequate ventilation/oxygenation in OR**

*Operational definition:* Inability to maintain PaO2 > 60 mm Hg (or SpO2 >90%) and / or PaCO2 > 60 mm Hg

*Code:* **ma12 laryngospasm**

*Operational definition:* Airway obstruction unrelieved by manuevers to relieve soft tissue obstruction and associated with SpO2 < 90%.

*Code:* **ma13 premature extubation**

*Operational definition:* Extubation of a patient prior to the return of upper airway protective reflexes, or the ability to maintain adequate oxygenation and ventilation.

*Code:* **mazz other airway occurrence**

*Operational definition:* Other airway-related occurrence.

*Code:* **mc00 Medical: Circulation or Cardiac**

*Operational definition:* Category for adverse occurrences related to circulation or cardiac systems.

*Code:* **mc01 air embolism**

*Operational definition:* Entrainment of air into the venous circulation and heart detected by any monitoring device including Doppler, TEE, or sudden decrease in end tidal CO2, SpO2, or blood pressure or air in coronary vessels on post-mortem exam

*Code:* **mc02 arrest**

*Operational definition:* Cardiac output insufficient to maintain a palpable central pulse, and requiring CPR, electroshock therapy and/or vasoactive drugs to maintain an adequate perfusion pressure.

*Code:* **mc03 arrhythmia(telemetry+Tx or mc06/death)**

*Operational definition:* Any cardiac rhythm which varies from baseline and requires either extra monitoring, drugs, consultations, or electroshock therapy, or results in hypotension or death.

*Code:* **mc04 CHF(new S3/JVD+rales/CXR+Tx)**

*Operational definition:* An abnormality of cardiac function is responsible for the failure of the heart to pump blood at a rate commensurate with the requirements of the metabolizing tissues, manifested by pulmonary edema, a new S3 gallop, jugular venous distension, rales, pleural edema or effusion, and requiring treatment.

*Code:* **mc05 hypertension**

*Operational definition:* Sbp > 180 or Dbp > 100 for > 5minutes

*Code:* **mc06 hypotension(sBP/MAP<50%base, >5min)**

*Operational definition:* Mean arterial pressure < 50% of baseline for > 5 minutes.

*Code:* **mc07 infarction(mc09+enzymes/new Qs)**

*Operational definition:* Necrosis of heart tissue as evidenced by elevated ST segments or new Q waves or new wall motion abnormality associated with elevated cardiac enzymes (troponin, CK-MB)

*Code:* **mc08 inappropriate or inadequate fluid therapy**

*Operational definition:* Insufficient replacement of volume with blood products, crystalloid or other colloid to maintain adequate perfusion and oxygenation of all tissues, as evidenced by inadequate urine output, low central filling pressures, elevated lactate, metabolic acidosis with pH < 7.35, and/or hypotension responsive to fluids. Criteria: (1) inadequate urine output (< 0.5 ml/kg/hr); (2) hypotension responsive to fluid challenge ; (3) elevated lactate level ; (4) metabolic acidosis (pH < 7.35); and/or (5) low central filling pressures.

*Code:* **mc09 ischemia(sx/1mmST 2 leads, ROMI/Tx)**

*Operational definition:* Myocardial ischemia is a deficiency of the blood supply to the heart muscle, leading to symptoms, flat depression of the ST segment of more than 0.1 mV below the baseline (i.e., the PR segment) and lasting longer than 0.08 s, treatment, or rule-out MI monitoring.

*Code:* **mc10 thermoregulation**

*Operational definition:* Temperature < 35oC for > 30min.

*Code:* **mczz other cardiac occurrence**

*Operational definition:* Other ciculation or cardiac-related occurrence.

*Code:* **md00 Medical: Drug**

*Operational definition:* Medication-related adverse occurrence.

*Code:* **md01 drug / allergic reaction**

*Operational definition:* The patient experienced an adverse reaction or an unsuspected allergic reaction to a medication.

*Code:* **md02 drug choice**

*Operational definition:* A medication was given to a patient with a known allergy or sensitivity, or an ineffectice drug was selected (e.g. antiobiotic was given after sensitivites showed organism to be resistant to it).

*Code:* **md03 drug dosage**

*Operational definition:* The dosage of a medication was ineffective or too large causing unwanted side effects such as hypotension, prolonged sedation, or treatment to reverse drug effects (e.g. narcan to coutnter act narcotics, or reversal of anticoagulation).

*Code:* **md04 drug interaction**

*Operational definition:* A medication adversely interacted with other drugs taken by the patient.

*Code:* **md05 drug neuromuscular block management**

*Operational definition:* Undesired residual neuromuscular blockade at the end of the procedure causing a delay in extubation.

*Code:* **mdzz other drug occurrence**

*Operational definition:* Other medication-related occurrence.

*Code:* **mg00 Medical: Gastrointestinal**

*Operational definition:* Category for adverse occurrences related to the gastrointentinal system.

*Code:* **mg01 ascites**

*Operational definition:* Effusion and accumulation of serous fluid in the abdominal cavity leading discernable on physical examination or radiologic imaging (free peritoneal fluid >25 ml), leading to symptoms, unplanned evaluation, or requiring treatment.

*Code:* **mg02 colitis**

*Operational definition:* Inflammation of the colon manifested as diarrhea or bloody diarrhea, sepsis, abdominal pain, or toxic megacolon. Criteria: 1. Rectal discharge; 2. Perineal ulceration; 3. Colonoscopic and biopsy evidence of inflammation.

*Code:* **mg03 GI bleeding(heme pos + drop Hct 10% or Tx)**

*Operational definition:* Blood loss through the gastrointestinal tract, including hematemesis, melena, hematochezia, occult GI bleeding may be identified in the absence of overt bleeding by special examination of the stool (e.g.,guaiac testing), or symptoms of blood loss or anemia such as lightheadedness, syncope, angina, or dyspnea.. Criteria: 1. Bloody vomitus or stool; 2. Bleeding from the rectum; 3. Hct decrease > 10%; 4. Lightheadedness, syncope, angina, or dyspnea.

*Code:* **mg04 ileus**

*Operational definition:* Abdominal distension and no passage of stool or flatus by postoperative day 3.

*Code:* **mg05 obstruction**

*Operational definition:* Pseudo-obstruction is colonic distension in the absence of mechanical obstruction, with cecal diam > 9 cm and air in all colonic segments on plain radiographs.

*Code:* **mg06 pancreatitis**

*Operational definition:* Acute inflammation of the pancreas with sudden onset of: (1) abdominal pain; (2) nausea; (3) vomiting; (4) high levels pancreas enzymes - serum amylase 3X normal.

*Code:* **mg07 perforation**

*Operational definition:* Iatrogenic perforation of the stomach, small intestine, or large intestine during the procedure or perforation later caused by implants or instrumentation. Criteria: (1) nausea, vomiting, or ileus; (2) abdominal or groin pain and referred pain; (3) air in the abdomen on plain radiograph or CT or other imaging study; (4) abdominal distension and tenderness; OR surgical finding of perforation.

*Code:* **mg08 peritonitis**

*Operational definition:* Inflammation or infection of the peritoneum with symptoms of: (1) abdominal pain and tenderness; (2) constipation; (3) vomiting; (4) moderate fever.

*Code:* **mgzz other GI occurrence**

*Operational definition:* Other GI-related occurrence.

*Code:* **mh00 Medical: Hematologic**

*Operational definition:* Hematologic adverse occurrence.

*Code:* **mh01 coagulopathy(INR>2 or Plts<50 or Fib<100)**

*Operational definition:* Any disorder reducing the ability of the blood to clot.

Severity 1: INR>_1.5 and < 2.0, or platelets <_100k and >50k

Severity 2: INR>_2.0 and < 3.0, or platelets <_50k and >20k

Severity 3: INR>_3.0, or platelets <_20k

*Code:* **mh02 DVT (confirmed by imaging)**

*Operational definition:* The presence of thrombosis of the iliac, femoral, or popliteal or other veins confirmed by imaging studies (duplex scan, CT, or MR) with or without swelling, warmth, erythema, or tenderness.

*Code:* **mh03 OR hemorrhage >3000cc**

*Operational definition:* Blood loss of greater than 3 L during the procedure.

*Code:* **mh04 transfusion occurrence**

*Operational definition:* The patient required an unplanned transfusion during or after the procedure, or advrse reaction to blood product transfusion.

*Code:* **mhzz other hematologic occurrence**

*Operational definition:* Other hematologic adverse occurrence.

*Code:* **mn00 Medical: Neurologic**

*Operational definition:* Category for adverse occurrences related to neurological function.

*Code:* **mn01 CVA/TIA(new focal deficit orCT/MR orBxAu)**

*Operational definition:* The abrupt onset of a nonconvulsive and new focal neurologic deficit due to a reduction of blood flow to the brain, or abnormality on imaging studies suggestive of a CNS infarct, or CNS infarction confirmed by biopsy or autopsy.

*Code:* **mn02 cerebral perfusion(ICP>20orCPP<30 for>5min)**

*Operational definition:* Reduction in the flow of blood to the brain during the procedure for > 5 minutes, with intracranial pressure >20 or cerebral perfusion pressure < 30 mmHg.

*Code:* **mn03 delerium(confusion>24h +Tx/sitter/restraint)**

*Operational definition:* Acute change in level of consciousness characterized by reduced ability to maintain attention to external stimuli, lethargy, or agitation, and disorganized thinking as manifested by rambling, irrelevant, or incoherent speech. Criteria: (1) confusion > 24 hr; and (2) was not related to narcotics; and (3) patient required restraints or continuous supervision.

*Code:* **mn04 diabetes insipidus**

*Operational definition:* Excessive urine production from reduced production or resonsiveness to ADH. Diagnosis can be made by relating plasma to urine osmolality, particularly in postoperative neurosurgical patients or after head trauma, where its use can permit quick differentiation of diabetes insipidus from parenteral fluid excess.

*Code:* **mn05 electrolyte change (Na<130/>150, K>5.5,other)**

*Operational definition:* The electrolyte balance of the extracellular fluid was sufficiently changed from normal to require extra monitoring, evaluation, or treatment beyond routine post-operative care. Specifically: Na < 130 or > 150 or K >5.5

*Code:* **mn06 meningitis(pos Cx/Bx or CT/MR and Tx)**

*Operational definition:* Inflammation of the meninges (the pia-arachnoid) and the cerebrospinal fluid (CSF) of the subarachnoid space associated with symptoms of fever, headache, nausea/diarrhea/abdmominal pain, and confirmed by CSF cultures or biopsy, imaging studies, and requiring treatment.

*Code:* **mn07 SAH/intracerebral hemorrhage**

*Operational definition:* Hemorrhage in the space between the arachnoid membrane and pia matter (subarachnoid) causing compression of the brain associated with sudden headache, neurological deifict, and confirmed with imaging studies or blood in the CSF. May also occur in the spinal cord in association with sudden back pain.

*Code:* **mn08 seizure**

*Operational definition:* A paroxysmal event due to abnormal, excessive, hypersynchronous discharges from an aggregate of central nervous system (CNS) neurons with manifestations ranging from convulsive activity to experiential phenomena not discernible by an observer, confirmed by EEG or neurology consultation.

*Code:* **mn09 withdrawal, alcohol(history + mn03 + Tx)**

*Operational definition:* A patient with history of alcohol abuse exhibits anxiety, confusion and delirium after the cessation of alcohol intake, requiring treatment.

*Code:* **mn10 withdrawal, narcotic**

*Operational definition:* The patient exhibits symptoms of nausea and diarrhea, coughing, lacrimation, mydriasis, rhinorrhea, profuse sweating, twitching muscles, and piloerection, or "goose bumps"; mild elevations in body temperature, respiratory rate, and blood pressure after reduction or cessation of narcotic intake, with improvement in symptoms after opiod administration.

*Code:* **mnzz other neurologic occurrence**

*Operational definition:* Other neurologic occurrence.

*Code:* **mr00 Medical: Respiratory**

*Operational definition:* Category for adverse occurrences related to the respiratory system.

*Code:* **mr01 ARDS(FiO2>50/vent>48h + mc04/mro5/BxAu)**

*Operational definition:* Acute hypoxemic respiratory failure due to pulmonary edema caused by increased permeability of the alveolar capillary barrier. Criteria: (1) FiO2 >50%; (2) Ventilator support for >48h; (3) PaO2/FiO2 <= 300 mm Hg; and (4) bilateral lung infiltrates on CXR.

*Code:* **mr02 empyema**

*Operational definition:* Purulent fluid collection in the pleural space confirmed by imaging studies and aspiration or by surgery.

*Code:* **mr03 hemothorax**

*Operational definition:* Blood in the pleural space confirmed by imaging studies and aspiration or surgery.

*Code:* **mr04 pleural effusion**

*Operational definition:* Pleural effusion is excess fluid in the pleural space.

*Code:* **mr04postop hypoxia(FiO2>50x48h or suppl O2x7d)**

*Operational definition:* Requirement for supllemental oxygen post-operatively, with FiO2 >50% for 48h or supplemental oxygen by nasal cannula for 7 days.

*Code:* **mr05 pneumonia(>38.0+Cx/CXR and Tx)**

*Operational definition:* Infection of the lung parenchyma confirmed by fever, sputum or brochial cultures, CXR, and requiring treatment.

*Code:* **mr06 pneumothorax**

*Operational definition:* Accumulation of gas in the pleural space resulting in symptoms (tachycardia, hypotension), requiring extra surveillance (e.g. repat CXRs or pulse oximetry) or treatment (chest tube placement).

*Code:* **mr07 pulmonary embolus(CTA/VQ/Angio + Tx)**

*Operational definition:* Sudden onset of shortness of breath, tachypnea, cyanosis, tachycardia, hypotension, or chest pain confirmed to be a imaging studies to be a pulmanry thrombus, and requiring treatment; or diagnosis made at autopsy.

*Code:* **mr08 respiratory arrest**

*Operational definition:* Sudden cessation of voluntary breathing, requiring CPR or mechanical ventilation.

*Code:* **mr09 other respiratory**

*Operational definition:* Other respiratory problem.

*Code:* **mu00 Medical: Urologic**

*Operational definition:* Category for adverse occurrences related to urological system.

*Code:* **mu01 Foley catheter trauma**

*Operational definition:* Injury to the urethra or bladder caused during normal insertion or removal of the Foley catheter, or during inadverdent removal of the catheter.

*Code:* **mu02 renal insufficiency (Cr >2 over base)**

*Operational definition:* Failure of the kidneys characterized by rapid decline in glomerular filtration rate (hours to days), retention of nitrogenous waste products, and perturbation of extracellular fluid volume and electrolyte and acid-base homeostasis. Criteria: serum Cr >2 above baseline.

*Code:* **mu03 urinary retention**

*Operational definition:* Inability to empty bladder under voluntary control.

*Code:* **mu04 UTI**

*Operational definition:* The presence of large amounts of bacteria (>100,000 organisms/mL) in the upper or lower urinary tract associated with symptoms or requiring treatment.

*Code:* **muzz other urologic event**

*Operational definition:* Other urologic adverse occurrence.

*Code:* **mv00 Medical: Visual**

*Operational definition:* Category for adverse occurrences associated with vision.

*Code:* **mv01 blindness (PION)**

*Operational definition:* Vision impairment, loss, or complete blindness associated with spine surgery either intraoperatively or postoperatively, and diagnosed by formal ophthalmologic examination.

*Code:* **mv02 corneal abrasion**

*Operational definition:* Damage to the corneal epithelium during the procedure caused by a foreign body or inadverdent contact.

*Code:* **mvzz other visual event**

*Operational definition:* Other visual system adverse occurrence.

*Code:* **pp00 Process occurrence**

*Operational definition:* Category for adverse occurrences related to coordination of care or administrative processes.

*Code:* **pp01 anesthesia team not called**

*Operational definition:* The anesthesia team was not notified about the planned procedure which resulted in delay of >20min.

*Code:* **pp02 surgical team not called**

*Operational definition:* The surgical team was not notified about the planned procedure which resulted in delay of >20min.

*Code:* **pp03 cancellation of procedure before incision**

*Operational definition:* The procedure was cancelled after the patient was brought down to the OR suite or pre-op area but before the incision was made.

*Code:* **pp04 implant or instrumentation defective**

*Operational definition:* The implant system or instrumentation system was discovered to be defective after inititaion and prior to conclusion of the procedure, resulting in delay of >20min during the procedure.

*Code:* **pp05 implant or instrumentation set incomplete**

*Operational definition:* The implant or instrumentation set was incomplete, delaying (>20min) or postponing the procedure.

*Code:* **pp06 implant or instrumentation unavailable**

*Operational definition:* The size or type of implant or instrumentation needed for the procedure was not available, resulting in delay (>20min) or postponement of the procedure, or the use of a suboptimal implant.

*Code:* **pp07 implant or instrumentation unfamiliar**

*Operational definition:* The procedure was delayed (>20min) or postponed because the surgical team was unfamiliar with the planned implant or instrumentation.

*Code:* **pp08 implant, wasted / wrong one opened**

*Operational definition:* An implant or graft was opened but not used, or the wrong size or type of implant was opened.

*Code:* **pp09 Pt to OR needing more tests**

*Operational definition:* The patient was transported to the OR before all required diagnostic tests were performed, resulting in delay of the procedure (>20min), postponement of the procedure, or adjustment/modification of the procedure.

*Code:* **pp10 Pt to OR without adequate consent**

*Operational definition:* The patient was transported to the OR suite or received sedative preop medications before informed consent was obtained, resulting in delay (>20min) or postponement of surgery, modification of the procedure, or requring consent from another source.

*Code:* **pp11 Pt to OR without surgical H&P**

*Operational definition:* The patient was transported to the OR suite without documention of adequate history and physical examination in the chart.

*Code:* **pp12 Pt to OR without test results**

*Operational definition:* The patient was transported to the OR suite before the results of all needed diagnostic tests were evaluated, resulting in delay (>20min) of the procedure, or modification of the procedure, or modification of monitoring during the procedure.

*Code:* **ppzz other process occurrence**

*Operational definition:* Other process or systems related adverse occurrence.

*Code:* **td00 Technical: Device**

*Operational definition:* Category for adverse occurrences related to surgical technique or surgical device.

*Code:* **td01 anesthetic equipment failure**

*Operational definition:* There was a failure in the anesthesia delivery equipment during the procedure causing an adverse outcome for the patient or a delay (>10min) during the procedure.

*Code:* **td02 cervical traction related occurrence**

*Operational definition:* Traction was used when contraindicated, or used improperly (e.g. overdistraction), or the incorrect traction device (e.g. non-MRI compatible tongs despite planned MRI) was used, or the traction device was improperly applied (e.g. incorrect position or pin torque).

*Code:* **td03 contamination of surgical field**

*Operational definition:* The sterile surgical field was violated during surgery.

*Code:* **td04 count difficulty, spinal level**

*Operational definition:* At any time prior to and during the procedure, the spinal levels was difficult to identify, or treatment was planned or initiated or performed for the wrong spinal level, with correction before completion of the procedure.

*Code:* **td05 count discrepancy, instr/sponge/needle**

*Operational definition:* There was a discrepancy between the preop and postop instrument, sponge, or needle counts.

*Code:* **td06 count discrepancy, spinal level**

*Operational definition:* The spinal procedure was performed at the wrong vertebral level or on the wrong side, without correction prior to completion of the procedure.

*Code:* **td07 failure to achieve reduction**

*Operational definition:* Failure of the treatment to correct or restore the fracture, dislocation, or deformity to expected, desired, or near anatomic position.

*Code:* **td08 failure of closed treatment**

*Operational definition:* Failure of fracture or soft tissue healing after nonoperative treatment, resulting in progressive symptoms or deformity AND requiring surgical intervention.

*Code:* **td09 fracture at / above / below implant**

*Operational definition:* The patient experienced a new fracture in the vertebrae at, superior, or inferior to the instrumented level(s).

*Code:* **td10 graft settling/displacement/dislodgement**

*Operational definition:* The graft or interbody cage moved downwards or laterally by more than 5mm from its position during surgery or immediately postoperatively.

*Code:* **td11 halo ring or frame related occurrence**

*Operational definition:* Defined as pin loosening, or pin site infection, slippage of the halo ring on the skull, pin site pain, pressure sores from the vest, dysphagia, more than expected scarring, bleeding, nerve injury, sinus puncture, or dural puncture, requiring additonal evaluation, treatment, or pin-repositioning.

*Code:* **td12 implant breakage**

*Operational definition:* The implant or instrumentation placed in the patient broke sometime the procedure.

*Code:* **td13 implant choice suboptimal**

*Operational definition:* The implant or instrumentation could not be installed according to the pre-operative plan, or it did not satisfactorily correct the problem, or it failed prior to discharge from the hospital.

*Code:* **td14 implant migration or loosening**

*Operational definition:* The implant moved greater than 5 mm in any direction post-operatively.

*Code:* **td15 implant placement suboptimal**

*Operational definition:* The implant was placed in a position where it doest not provide proper function (e.g. alignment or fixation), or where it could damage vessels, nerves or organs, and leading to additonal monitoring, imaging, or treatment (e.g. repositioning).

*Code:* **td16 line failure, arterial**

*Operational definition:* The arterial line stopped working during the surgery or was inadvertently dislodged, requiring interruption of surgery for replacement or alternate monitoring.

*Code:* **td17 line failure, central**

*Operational definition:* The central venous line stopped working during the surgery or was inadvertently dislodged, requiring interruption of surgery for replacement or alternate access.

*Code:* **td18 line failure, infiltration**

*Operational definition:* One of the lines resulted in extravasation of fluid into the surrounding tissues.

*Code:* **td19 line failure, iv disconnect**

*Operational definition:* An IV line stopped working during the surgery or was inadvertently dislodged, requiring interruption of surgery for replacement or alternate access.

*Code:* **td20 line placement, arterial**

*Operational definition:* There was difficulty with the arterial line that required delay (>20min).

*Code:* **td21 line placement, central**

*Operational definition:* There was difficulty placing the central venous line causing delay in surgery of >20min.

*Code:* **td22 loss of spinal alignment**

*Operational definition:* At post-operative evaluation the patient has clearly greater deformity than initially present after surgery (change of more than 5 degrees or more than 5mm).

*Code:* **td23 retained foreign body**

*Operational definition:* A surgical instrument, sponge, needle, or unused implant (instrumentation) was left inside the patient after closing the incision.

*Code:* **td24 surgical positioning occurrence**

*Operational definition:* The equipment used for maintaining the patient in the desired surgical position interfered with other surgical equipment, delayed surgery by more than 20min, interrupted surgery, or caused an injury ot the patient.

*Code:* **tdzz other technical occurrence**

*Operational definition:* Other technical or device related adverse occurrence.

*Code:* **ti00 Technical: Injury**

*Operational definition:* Category for adverse occurrences related to an injury associated with the surgical procedure or its technical performance.

*Code:* **ti01 awareness or recall**

*Operational definition:* The patient could recall intraoperative events after awakening from the procedure.

*Code:* **ti02 bladder injury**

*Operational definition:* The bladder was inadvertently injured during the procedure (excluding Foley catheter trauma mu01 and neural injury ti15), recognized during or after the procedure.

*Code:* **ti03 bowel injury**

*Operational definition:* The large or small intestine was inadvertently perforated during the injury, recognized during or after the procedure.

*Code:* **ti04 burn (thermal) injury**

*Operational definition:* Skin, vascular structures or organs were inadvertently burned during surgery, recognized during or after the procedure.

*Code:* **ti05 dental or denture injury**

*Operational definition:* The teeth, gums, or dental work were inadvertently damaged during the procedure.

*Code:* **ti06 employee exposure, body fluid**

*Operational definition:* A member (or members) of the surgical team had skin or mucous membrane exposure to patient's blood, body fluid, or respiratory secretions.

*Code:* **ti07 employee exposure, sharp**

*Operational definition:* A member (or members) of the surgical team had their skin broken by a scalpel, needle, bone, or other sharp object, resulting in exposure to blood, body fluid, or respiratory secretions or the patient or the caregiver.

*Code:* **ti08 esophageal injury**

*Operational definition:* The esophagus was injured during the procedure, recognized during or after the procedure.

*Code:* **ti09 monitoring, EMG neurotonic activity**

*Operational definition:* The electrophysiologist recorded abnormal EMG activity (sustained or neurotonic discharges) during the procedure suggesting possible neural injury, leading to interruption of the surgery, change in surgical activity, additonal diagnostic evaluation during or after surgery (e.g. imaging), or additonal treatment (e.g. sterioids).

*Code:* **ti10 monitoring, SSEP decrease >50%**

*Operational definition:* The electrophysiologist recorded changes sensory or motor eveoked potentials suggesting neural injury, leading to interruption of the surgery, change in surgical activity, additonal diagnostic evaluation during or after surgery (e.g. imaging), or additonal treatment (e.g. sterioids).

*Code:* **ti11 muscle, compartmental syndrome**

*Operational definition:* Increased tissue pressure caused by edema in the paraspinal muscle compartment compromised the neuromuscular and vascular function of the muscles, leading to additonal monitoring or treatment (e.g. compartmental release) or late clinical maifestations.

*Code:* **ti12 neural, erectile dysfunction**

*Operational definition:* The patient experienced impairment in erectile function after the procedure (excluding retrograde ejaculation).

*Code:* **ti13 neural, intraoperative root injury**

*Operational definition:* The patient sustained an inadverdent nerve root injury recognized during the procedure (with or without clinical consequences).

*Code:* **ti14 neural, laryngeal nerve injury/hoarseness**

*Operational definition:* The patient experienced a laryngeal nerve injury during the procedure causing hoarsness or vocal cord dysfunction lasting until discharge from hospital or more than 2 weeks after surgery.

*Code:* **ti15 neural, new cord / cauda equina injury**

*Operational definition:* The patient experienced a new spinal cord or cauda equina deficit lasting until discharge from hospital or more than 2 weeks after surgery.

*Code:* **ti16 neural, new radiculopathy**

*Operational definition:* After the procedure, the patient developed a new sensory (noticeable by the patient) or motor deficit (change of 2 or more grades) that follows the distribution of a specific nerve root, lasting until discharge from hospital or more than 2 weeks after surgery.

*Code:* **ti17 neural, pressure palsy**

*Operational definition:* The patient experienced a compressive injury to a nerve plexus during the procedure leading to symptoms of numbness, dysesthesia, or weakness lasting until discharge from hospital or more than 2 weeks after surgery.

*Code:* **ti18 neural, retrograde ejaculation**

*Operational definition:* After the procedure, the patient experienced new onset of loss of ejaculation (the semen diverted up the urethra towards the bladder during ejaculation).

*Code:* **ti19 neural, worsening of cord injury**

*Operational definition:* The patient experienced worsening of a pre-existing spinal cord or cauda equina injury at any time after the procedure, with worsening lasting until discharge from hospital or more than 2 weeks after surgery.

*Code:* **ti20 neural, worsening of radiculopathy**

*Operational definition:* The patient experienced worsening of a pre-existing radiculopathy at any time after the procedure, with worsening lasting until discharge from hospital or more than 2 weeks after surgery.

*Code:* **ti21 pressure sore, brace or cast**

*Operational definition:* The patient developed a pressure sore from the brace or cast requirng change in brace, discontinuation of brace, treatment of pressure sore, treatment of symptoms from pressure sore, or leading to scar formation.

*Code:* **ti22 pressure sore, face/head/pressure alopecia**

*Operational definition:* The patient developed a pressure sore on the face or head or loss of hair caused by positioning during the procedure or treatment requirng treatment of pressure sore, treatment of symptoms from pressure sore, or leading to scar formation.

*Code:* **ti23 pressure sore, sacral**

*Operational definition:* The patient developed pressure sores on the lower back requirng treatment of pressure sore, treatment of symptoms or infection from pressure sore, or leading to scar formation.

*Code:* **ti24 pressure sore, other body area**

*Operational definition:* The patient developed pressure sores other than due to casting or bracing (ti21), head positioning (ti22), or supine positioning (ti23), requiring treatment of pressure sore, treatment of symptoms from pressure sore, or leading to scar formation.

*Code:* **ti25 swallowing difficulty**

*Operational definition:* Patient experienced difficulty swallowing and eating solid foods at any time after the surgical procedure, lasting until discharge from hospital or for at least 2 weeks after surgery.

*Code:* **ti26 ureteral injury**

*Operational definition:* The ureter was was inadvertently injured during the procedure (excluding Foley catheter trauma mu01 and neural injury ti15), recognized during or after the procedure.

*Code:* **ti27 vessel injury, artery**

*Operational definition:* An artery was inadvertantly injured during the procedure requiring repair or post-operative monitoring (e.g. vascular checks or duplex) or imaging (duplex or CTA or angiography).

*Code:* **ti28 vessel injury, vein**

*Operational definition:* A vein was inadvertantly injured during the procedure requiring repair or post-operative monitoring (e.g. vascular or compartment checks or duplex scan) or imaging (duplex or CTA or angiography).

*Code:* **ti29 pseudomeningocele**

*Operational definition:* Imaging studies are consistent with CSF collection adjacent to dura and the patient reports symptoms of postural headache or visual disturbace or associated nerve root pain.

*Code:* **ti30 unintended dural opening**

*Operational definition:* The dura was inadvertently opened during the procedure, with CSF extravasation or bulging of the arachnoid layer.

*Code:* **ti31 unintended pleural/peritoneal opening**

*Operational definition:* The pleural or peritoneal membranes were unintentionally violated during the procedure.

*Code:* **tizz other technical occurrence**

*Operational definition:* Other surgical technique related injury or adverse occurrence.

*Code:* **wh00 Wound Healing and Infection**

*Operational definition:* Category for adverse occurrences related to wound healing or post-operative infection.

*Code:* **wh01 abscess, epidural**

*Operational definition:* The patient developed a localized collection of pus outside the dura after surgery at that level. Criteria: (1) fever >38,0 deg C OR back or neck pain OR local tenderness OR Neurologic deficit AND (2) CT myelo/MRI shows epidural fluid collection OR culture is positive on biopsy AND (3) requires treatment with antibiotics and/or surgery.

*Code:* **wh02 abscess, paraspinal(wh09 +CT/MR/OR)**

*Operational definition:* The patient developed a localized collection of pus in the paraspinal muscle compartment after surgery. Criteria: (1) fever >38,0 deg C OR back or neck pain OR local tenderness OR Neurologic deficit AND (2) CT myelo/MRI shows paraspinal fluid collection OR culture is positive on biopsy AND (3) requires treatment with antibiotics and/or surgery.

*Code:* **wh03 bacteremia(Blood Cx + Tx)**

*Operational definition:* The patient developed bacteria following the procedure, confirmed with positive blood cultures, and requiring treatment, but no symptoms of sepsis.

*Code:* **wh03 CSF leak**

*Operational definition:* The patient developed a leak of cerebrospinal fluid from the skin incision after the procedure, requiring additonal diagnostic tests, or requiring prolongation of wound care or bed rest or antibiotics, or requiring lumbar drain placement, or requiring repeat surgery. Criteria: (1) clear liquid draining from incision with beta-transferrin present in fluid or CSF symptoms (postural headache OR posterior neck pain or stiffness OR nausea OR photosensititivty OR visual symptoms OR tinnitus OR vertigo); AND (2) paraspinal spinal fluid collection on imaging studies; AND (3) requires treatment (bed rest, drain, or surgery).

*Code:* **wh04 drainage, prolonged**

*Operational definition:* The patient had fluid issuing from incision for longer than 7 days after surgery.

*Code:* **wh05 dehiscence**

*Operational definition:* The patient's incision split open after surgery, requiring open wound care or repeat surgery.

*Code:* **wh06 fever, unknown etiology(>38.0/d x3d)**

*Operational definition:* The patient experienced a fever (>38.0 deg C) prolonging hospitalization or lasting at least three consecutive days, with no identified source after diagnostic evaluation.

*Code:* **wh07 hematoma, wound/epidural(required OR)**

*Operational definition:* The patient had symptoms of pain and swelling (no fever or erythema) at the surgical incision, or developed hemorrhagic drainage from the incision, or developed neurological deficit, with imaging studies showing fluid collection in the surgical field, or patient required repeat surgery for exploration or evacuation of a possible epidural hematma.

*Code:* **wh08 sepsis(mc06+wh09 or mc06+Cx/Bx/Xray)**

*Operational definition:* The patient developed hypotension (mc06) and bacteremia in association with deep wound infection (wh09), confirmed by cultures and imaging studies or surgery.

*Code:* **wh09 wound infection, deep(wh10 + wh05/OR)**

*Operational definition:* The patient developed an infection at the surgical site requiring treatment. Criteria: (1) fever >38,0 deg C; and (2) increased incisional pain, erythema, tenderness, or drainaige or culture/stain is positive on biopsy; AND (3) requires treatment with antibiotics plus surgery (deep).

*Code:* **wh10 wound infect, superficial(drainage/Cx +Tx)**

*Operational definition:* The patient developed an infection at the surgical site requiring treatment. Criteria: (1) fever >38,0 deg C; and (2) increased incisional pain, erythema, tenderness, or drainaige AND (2) requires treatment with antibiotics only (superficial).

*Code:* **zh00 Late**

*Operational definition:* Category for adverse occurrences that generally occur sometime after surgery, generally more than 3 months following surgery.

*Code:* **zh01 deformity**

*Operational definition:* The patient's spinal alignment deteriorated relative to the immediate postoperative alignment, leading to symptoms, impaired function, >30 degrees scoliosis, >30 degreess kyphosis, or requiring additional treatment.

*Code:* **zh02 instability**

*Operational definition:* The patient developed excessive motion (>5 degrees rotation or 5mm translation greater than the amount expected) at a vertberal level within or adjacent to the surgical site, leading to neural deficit, deformity, or pain, and requiring additonal evaluation or treatment.

*Code:* **zh03 junctional arthrosis**

*Operational definition:* The patient developed arthritic changes at facet joints or disc spaces at the level above or the level below spinal fusion, leading to symptoms requiring additonal treatment (activity restriction, bracing, or surgery).

*Code:* **zh04 nonunion**

*Operational definition:* At more than 6 months after an arthrodesis, the bone grafts failed to integrate with the patients native bone, preventing the formation of a solid fusion mass, leading to symptoms (pain or defornmity) and hardware failure (breakage or loosening) or revision surgery. Criteria: At more than six months after fusion: (1) radio-lucent line (wider than 2mm) at the graft-vertebral body interface OR no trabeculae bridging the graft-vertebral body interface or settling (>5mm) of the graft; AND (2) more than 5 degrees of motion on F/E radiographs OR implant migration OR implant breakage OR other deformity; AND (3) requires treatment with repeat surgery, bracing, or other measures (e.g. external magnetic stimulation).

*Code:* **zhzz other late occurrence**

*Operational definition:* other late occurrence.
